# Out of the core: the impact of focal ischemia in regions beyond the penumbra

**DOI:** 10.3389/fncel.2024.1336886

**Published:** 2024-03-05

**Authors:** Ludmila Koukalova, Martina Chmelova, Zuzana Amlerova, Lydia Vargova

**Affiliations:** ^1^Department of Neuroscience, Second Faculty of Medicine, Charles University, Prague, Czechia; ^2^Department of Cellular Neurophysiology, Institute of Experimental Medicine of the Czech Academy of Sciences, Prague, Czechia

**Keywords:** stroke, remote areas, astrocyte, microglia, NG2-glia, oligodendrocytes, therapy, future outlooks

## Abstract

The changes in the necrotic core and the penumbra following induction of focal ischemia have been the focus of attention for some time. However, evidence shows, that ischemic injury is not confined to the primarily affected structures and may influence the remote areas as well. Yet many studies fail to probe into the structures beyond the penumbra, and possibly do not even find any significant results due to their short-term design, as secondary damage occurs later. This slower reaction can be perceived as a therapeutic opportunity, in contrast to the ischemic core defined as irreversibly damaged tissue, where the window for salvation is comparatively short. The pathologies in remote structures occur relatively frequently and are clearly linked to the post-stroke neurological outcome. In order to develop efficient therapies, a deeper understanding of what exactly happens in the exo-focal regions is necessary. The mechanisms of glia contribution to the ischemic damage in core/penumbra are relatively well described and include impaired ion homeostasis, excessive cell swelling, glutamate excitotoxic mechanism, release of pro-inflammatory cytokines and phagocytosis or damage propagation via astrocytic syncytia. However, little is known about glia involvement in post-ischemic processes in remote areas. In this literature review, we discuss the definitions of the terms “ischemic core”, “penumbra” and “remote areas.” Furthermore, we present evidence showing the array of structural and functional changes in the more remote regions from the primary site of focal ischemia, with a special focus on glia and the extracellular matrix. The collected information is compared with the processes commonly occurring in the ischemic core or in the penumbra. Moreover, the possible causes of this phenomenon and the approaches for investigation are described, and finally, we evaluate the efficacy of therapies, which have been studied for their anti-ischemic effect in remote areas in recent years.

**Key points**

• The majority of clinical and experimental research of stroke focuses on the most severely affected area, the ischemic core, and the tissue with recovery potential, the penumbra, while the other brain structures are often neglected.
• The remote areas, even seemingly undamaged, can experience delayed impairment after the initial injury in the core, with a potentially deteriorating functional impact—the phenomenon called diaschisis.
• While morphological and functional alterations of glia are frequently observed in the remote areas, their contribution to mechanisms of damage propagation into the remote areas is not yet fully elucidated.
• The dual nature of many glial functions allows them to play a crucial role in mitigating/preventing tissue damage but also to exacerbate inflammation and excitotoxicity, thus highlighting the complexity of their participation in the nervous system damage and recovery.

## 1 Introduction

Ischemic stroke has been studied extensively due to its pervasive nature on patients’ quality of life and economic burden on the public health care system, nevertheless, the current available treatment provides less than satisfactory results (French et al., 2016). In clinical medicine as well as in the experimental research, close attention is usually given to the region with the greatest destruction, the ischemic core, as well as to the tissue with recovery potential, the penumbra (Saver, 2017). This focus on the parts that are affected the most seriously is understandable since those areas undergo the most noticeable alterations. However, the understanding of ischemic mechanisms would not be complete if we only considered the most severely damaged regions, since the seemingly undamaged brain regions may also fall victim to the secondary injury. This process was assigned as exo-focal neuronal death (Zhao et al., 2002) (although neurons are certainly not the only ones affected, see below) or more generally diaschisis, describing the phenomenon where transient malfunction of a distant area occurs as a result of local brain injury (Yang et al., 2013). Ischemic stroke is not the only condition linked to diaschisis – it has been described in cases of epilepsy, migraine, encephalitis, brain tumors and traumatic brain injuries (Paradowski and Pawlik, 2005; Poretti and Boltshauser, 2012), i.e., generally with pathologies associated with inflammation, glia activation, spreading depolarization, apoptosis, oxidative stress and ionic imbalance in the intracellular or extracellular space (ECS).

Even though the site of injury is demarcated by a barrier comprised of cells and fibrous material (Wang et al., 2018) partially preventing the damage from spreading, ischemia also poses a tremendous burden on the healthy surrounding tissue, since not all defensive mechanisms can be engaged at once (Shi et al., 2019). For instance, depolarization elicited in the penumbra do not dissipate at the border with healthy tissue but propagate further (Andrew et al., 2022). The effect of ischemia can then be manifested in the remote areas in the similar way as in the core only in a less profound intensity or reversed manner (Arvidsson et al., 2001; Li et al., 2020). Nevertheless, the changes in remote areas have one thing in common – they appear with a delay after the initial reaction in the core (Zhang et al., 2012; Li et al., 2020). An assessment of whether the damage in remote areas may participate in the final behavioral or cognitive deficit after a stroke attack may be rather difficult for several reasons. First, the association of an injury to a specific brain region with a neurological deficit is complex, and the regional damage may not correspond with the typical behavioral and/or cognitive outcome (Zhou et al., 2013). Second, the disturbance of mental functions can be orchestrated by different parts of the brain, just as normal body functions are not controlled solely by one brain structure (Poldrack, 2010). Moreover, new areas can adopt the role of the damaged region after stroke (Gerloff et al., 2006). Thus, it is reasonable to assume that the state of remote regions matters; certainly the evidence supports this presumption. Several studies have reported a significant correlation between a decrease in regional blood flow and metabolism in the remote cerebellum with clinical stroke scales (Liu et al., 2007; Szilagyi et al., 2012; Shinohara et al., 2017; Wang et al., 2020; Chen et al., 2022). Interestingly, Takasawa M. et al. (2002) were able to obtain such results in the subacute post-ischemic stage, when the changes in remote areas start to manifest, but not in the acute phase (Takasawa M. et al., 2002). Alternatively, we can look at the problem from another point of view: the hippocampus is usually spared from ischemic insult, as the blood supply is not provided by the middle cerebral artery (MCA) (Rusinek et al., 2011), which is a blood vessel frequently occluded during a stroke (Rovito et al., 2021). Therefore, the hippocampus is often considered a region remote from the ischemic core (Gulyaeva et al., 2021). However, depression and dementia are common post-stroke complications that are associated with impaired function of the hippocampus, which could be regarded as intact at first sight (Onufriev et al., 2021). The distant areas may thus play an important role in the clinical outcome. Moreover, in contrast to the rapidly damaged core, we can take advantage of the delayed exo-focal reaction and preserve the still intact remote tissue by using a suitable intervention (Kidani et al., 2020).

In addition to neurons, glial cells are also affected and responsive to ischemic injury (Mihailova et al., 2023). Due to their multiple functions and the large number of released cytokines/chemokines, glial cells often have a “dual face” as they can play an irreplaceable role in mitigating/preventing tissue damage, but they also exacerbate inflammation and excitotoxicity (Pekny et al., 2014; Quincozes-Santos et al., 2021). Therefore, glia-oriented therapies aimed at the detrimental functions of glia, that can intensify or propagate ischemic injury, progressively prevail over inefficient neuron-centered approaches in the preclinical phase [for review see (Hernandez et al., 2021)]. The glia-related mechanisms, such as neuroinflammation, reversed glutamate uptake, extracellular matrix (ECM) remodeling, disruption of myelin sheets and calcium waves are already generally accepted as contributors of ischemic injury, spreading from the core to the penumbra. However, their involvement in the propagation of the ischemic insult into the remote areas remains hypothetical and has yet to be fully elucidated. In this review, using the research data published in the last 20 years, we present comprehensive information regarding the impact of focal ischemia on brain tissue outside of the penumbra. We discuss in particular the post-ischemic reaction of neurons, glial cells and ECM in various brain regions and the possible therapeutic approaches. Supplementary Table 2 provides a concise summary.

## 2 Glial cells and their role in ischemic injury

Although glial cells were originally described by Virchow’s and other early studies as purely supportive elements in the brain (Chvatal and Verkhratsky, 2018), years of intensive research revealed their multifaceted function in development and physiological conditions, as well as in various central nervous system (CNS) injuries and diseases, including ischemia (Verkhratsky, 2007; Allen and Lyons, 2018; Mihailova et al., 2023). Here, we briefly introduce the most well known and most studied glial cell types in ischemia - astrocytes, microglia, oligodendrocytes and NG2-glia. The following chapters will discuss more detailed information, focusing on the structural/functional alterations and localization of individual glial cell types in post-ischemic tissue and the distinct remote areas, and their participation in ischemic damage and propagation.

**Astrocytes**, the most prevalent type of glial cells, as an important component of the blood-brain barrier (BBB), can improve nutritional support for neurons by regulating the capillary blood flow and releasing ketone bodies that supply energy. In addition, the astrocytic uptake of excitotoxic substances, mainly glutamate, by transporting proteins present in their plasma membrane, helps to restrict the spread of damage to the surrounding environment (Hernandez et al., 2021). Furthermore, astrocytes also help maintain a stable pH and ionic homeostasis by removing the protons and potassium released in the ECS during neural activity (Gradisnik and Velnar, 2023). Ischemia-induced injury triggers astrocyte activation, including changes in protein expression (e.g., upregulation of glial fibrillary acidic protein, GFAP), and morphological changes, such as branching levels and length of processes (Pekny and Nilsson, 2005). These morphological changes are dependent on the distance from the infarction area (Li et al., 2022). Based on their gene expression and consequent role, activated astrocytes can be divided into 2 main types: A1 (inflammation-induced, pro-inflammatory) and A2 astrocytes (ischemia-induced, anti-inflammatory) (Zamanian et al., 2012; Liddelow et al., 2017). The A1 type is considered harmful, releasing pro-inflammatory mediators (e.g., interleukin IL-6, or tumor necrosis factor α, TNF-α) and acting detrimental to synapses. The A2 type release anti-inflammatory compounds and growth factors, such as brain-derived neurotrophic factor (BDNF) and promote the survival and growth of neurons (Liddelow and Barres, 2017; Xie and Liu, 2023). However, the development of advanced transcriptomic analyses, including single-cell RNA sequencing, has led to an accumulation of evidence that there are multiple types of reactive astrocytes. For example, in post-ischemic penumbra, 7 subgroups of astrocytes were identified (Guo et al., 2021).

Astrocytic glutamate uptake in ischemia can be reversed and thus enhances excitotoxic damage (Gouix et al., 2009). Moreover, ischemia-evoked astrocytic swelling is accompanied by compensatory ECS shrinkage that further increases the concentration of potentially toxic agents (Sykova, 2004; Lafrenaye and Simard, 2019). In addition, astrocytes contribute to the BBB breakdown by detaching their endfeet from capillaries and producing substances that promote blood vessel permeability [e.g., vascular endothelial growth factor (VEGF), matrix metalloproteinases (MMPs), nitric oxide (NO), and endothelin-1] (Zhang et al., 2020). Structural alterations in reactive astrocytes and overproduction of ECM hinder the flow of growth hormones and neuroactive substances across the ECS, impairing their potential regeneration as well as extrasynaptic intracellular communication (Vargova and Sykova, 2014; Wang et al., 2018). Moreover, astrocytes coupled by gap-junctions create a syncytium, which, by the spread of calcium waves and ATP release, enables the so-called gliotransmission and distant neuro-glia communication. Under ischemic conditions, these mechanisms allow the propagation of the injury into the neighboring tissue and may thus affect the cells in remote areas (Verkhratsky, 2007).

**Microglia** are mainly “cleaning agents”, that eliminate potentially harmful substances as well as dysfunctional synapses and thus mediate tissue remodeling. In addition, in later periods of post-ischemic regeneration, they release a variety of neuroprotective factors (Xu S. et al., 2020). Both microglia and astrocytes produce pro-inflammatory cytokines when activated by ischemia, and the dysregulated inflammatory response can worsen the functional and tissue damage in the ischemic brain (Xu S. et al., 2020). Activation of microglia includes a change in gene expression [e.g., upregulation of ionized calcium-binding adapter molecule 1 (Iba-1), and a cluster of differentiation 68 (CD68)] as well as microglial polarization (Ito et al., 2001; Perego et al., 2011). Similarly to astrocytes, the phenotype of activated microglia can be either the pro-inflammatory M1 type or the anti-inflammatory M2 type. The M1 phenotype produces inflammatory cytokines and chemokines, such as TNF-α, IL-1β, or interferon-γ (IFN-γ), and promotes neuronal death. The M2 acts as a beneficiary phenotype, releasing anti-inflammatory compounds (IL-10, IL-4, or transforming growth factor TGF-β) and neurotrophic factors (Lee et al., 2014). In the early stages of ischemia, M2 predominates over the M1 type. Later, the microglial phenotype is gradually shifted toward the M1 type in peri-infarct regions (Hu et al., 2012). However, more recent studies using gene expression profiling showed that the division of microglia into only 2 types is not sufficient. Using single-cell RNA-sequencing, Guo et al. (2021) found 14 microglial subgroups, which showed significant variability in expression profiles and uneven distribution between the ischemic middle cerebral artery occlusion (MCAO) and the control group (Guo et al., 2021).

**Oligodendrocyte progenitor cells**, also known as polydendrocytes or **NG2-glia**, exhibit a high proliferative and differentiation ability, primarily in myelinating oligodendrocytes under physiological conditions (Zhu et al., 2008; Dimou and Gallo, 2015). Under ischemic conditions, the number of NG2-glia was significantly decreased in the infarct core but significantly elevated in the penumbra (Tanaka et al., 2001). NG2-glia contribute to glial scar formation and wound closure, regulate neuroinflammation and are endowed with a high proliferative ability (Valny et al., 2017). Interestingly, under ischemic conditions, NG2-glia differentiate rather into reactive astrocytes than into oligodendrocytes, as evidenced by the immunohistochemical and electrophysiological properties of glia cells in ischemia (Honsa et al., 2016).

**Oligodendrocytes** are myelinating cells that sustain and insulate axonal myelin sheaths. Ischemia, accompanied by oxidative stress or excitotoxicity has a detrimental effect on oligodendrocytes, leading to their apoptosis and demyelination, which can have a significant impact on the neurological functions and final outcome of ischemia (Dewar et al., 2003).

**Tissue response to ischemic injury** comprises several complex mechanisms where glial cells can play pro-active or suppressive roles. These mechanisms include neuroinflammation (Shen et al., 2023), edema (Gu et al., 2022), oxidative stress (Radak et al., 2014), excitotoxicity (Kirdajova and Anderova, 2020), and glial scar formation (Silver and Miller, 2004; Kawano et al., 2012; Manrique-Castano and ElAli, 2021). The vast majority of these processes are considered detrimental, aggravating and expanding the tissue damage. However, the formation of a glial scar stands out among these reactions to ischemic/traumatic insult, as it assumes a dual role in the injured CNS. In the early stages of glial scar formation, reactive astrocytes release BDNF and suppress inflammation, protecting nerve cells from further damage (Rolls et al., 2009). In addition, scar formation also creates a barrier by depositing fibrotic molecules at the injury site, thus impeding neurotoxic substances, peripheral leukocytes and inflammatory signals to enter healthy tissue (Manrique-Castano and ElAli, 2021). Altogether, these actions safeguard neural tissue from propagating traumatic or pathological insults. While contributing to certain tissue protection, glial scar formation has negative consequences as well. They appear especially in later post-ischemic stages and include hindering the reconstruction of the BBB or preventing the promotion of axonal growth. Moreover, due to the release of pro-inflammatory cytokines, glial scar contributes to persistent widespread inflammation, promoting tissue degeneration (Kawano et al., 2012; Zhang et al., 2020; Manrique-Castano and ElAli, 2021). Interestingly, Zbesko et al. (2018) suggested that glial scar does not completely isolate the damaged area from healthy tissue but is partially permeable to toxic compounds contained in extracellular fluid released from the “area of liquefactive necrosis” (Zbesko et al., 2018).

## 3 Definition of the terms ischemic core, penumbra, and remote areas

Within the first several hours following the ischemic insult, three affected areas can be recognized: (1) the ischemic core; (2) the penumbra; (3) the remote areas.

**The core** may be defined as a region with a decrease of regional blood flow below 35 % in the grey matter and below 25 % in the white matter (WM) in human brain (Rodriguez-Vazquez et al., 2022). Hartings et al. (2017) claim the core can be best described as an area of persistent depolarization with the regional blood flow failing to reach 5 – 10 ml/100 g/min (Hartings et al., 2017).

**The penumbra** was initially characterized in a monkey model of stroke as tissue undergoing progressive damage surrounding a uniform central core destined for infarction (Astrup et al., 1977, 1981; Symon, 1980). The “tissue at risk” concept of the penumbra was based on intensive research in experimental animal models of stroke (Ebinger et al., 2009). The development of imaging techniques such as positron emission tomography (PET), computing tomography (CT) and magnetic resonance imaging (MRI) allowed visualization of the penumbra and helped to verify this concept also in humans, with varying core and penumbra definitions according to the used technique (Ermine et al., 2021). The penumbra is also deemed a salvageable area if prompt reperfusion is achieved (Witte et al., 2000). Interestingly, based on numerous studies involving animal models and stroke patients, del Zoppo et al. (2011) have suggested that both the infarct core and the ischemic penumbra exhibit heterogeneity in the early minutes and hours after ischemia. Their model consists of “mini-cores” surrounded by multiple “mini-penumbras”; without intervention, these “mini-penumbras” will be consumed by expanding “mini-cores”, and consequently encompassing larger region of injury (Jones et al., 1981; Tagaya et al., 2001; del Zoppo et al., 2011). In the non-reperfused penumbra, the apoptosis of still viable cells is induced, which leads to the spread of the ischemic core over a period of several hours (Liu et al., 2010; Genova, 2011; Saver, 2017). The crucial roles in the shift from reversible to irreversible tissue damage play spreading depression-like depolarization and excitotoxicity (Hartings et al., 2017; Sueiras et al., 2021; Andrew et al., 2022). This is one of the reasons why some researchers consider the precise division of ischemia-affected parts outdated (van Putten et al., 2021). However, several clinical studies confirmed that rescuing the penumbra in patients with a convenient core/penumbra imaging profile can considerably extend the therapeutical time window for thrombolytic therapy (Ermine et al., 2021).

**The remote areas** have been defined as regions undergoing tissue transformation, without cellular death (Karetko-Sysa et al., 2011), however, this is not always the case (Uchida et al., 2010; Park et al., 2011). According to some studies, the core is the only area of the affected regions where structural changes occur, whereas the remote structures undergo solely functional alterations (Poretti and Boltshauser, 2012; Yang et al., 2013). Nevertheless, there is a body of research that shows the opposite can be true; readers can find various examples of post-ischemic alterations in cell morphology, and a number of synapses or protein accumulations in this article.

The controversies between the studies arise mostly from the vague and subjective spatial/temporal definition of remote areas. Certainly, to indicate precisely the spatial characteristics of remote areas is almost impossible since it depends on the location, severity and mainly the duration of the vessel occlusion. However, there are some conditions that should be fulfilled in the “proper” remote areas. First of all, the vessels supplying the ischemic and remote areas differ, hence the remote areas are not directly affected by the initial deprivation of the blood flow but may undergo secondary changes or damage due to various processes triggered by the ischemic event (Karetko-Sysa et al., 2011). Second, the remote areas are usually located in a different brain structure than the ischemic core; in the case of the cortex, it should be a different lobe or even contralateral hemisphere (Bonilha et al., 2014). Third, there should exist functional connections between the core and the remote area. The temporal definition is slightly easier as the first changes in cell structure and functions in the core or penumbra can occur within minutes after ischemic onset (cytotoxic swelling), rapidly develop within hours (cell death) and the first week (glia activation and proliferation, ECM alterations, development of glial scar) and stabilize several months after ischemia (permanent glial scar formation or recovery in the case of reperfused penumbra). In contrast, the first changes in the remote areas can be detected within days or even months after stroke. Moreover, these alterations are mostly functional while structural changes in the remote areas are subtler than in the core or penumbra or they may be none.

**FIGURE 1:**
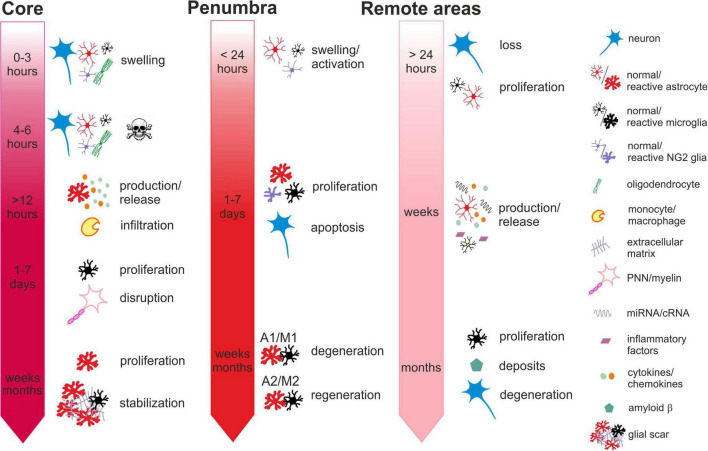
Time-dependent ischemia-induced changes in the ischemic core **(left)**, penumbra **(middle)** and remote areas **(right)**. *Core:* Profound cell swelling occurs several minutes after the onset of ischemia. Within 6 hours, neurons undergo necrosis and general loss of cells can be observed. Around 12 hours after ischemia, the surviving astrocytes begin to release chemokines attracting migration and infiltration of monocytes/macrophages. One to seven days after ischemia, the number of cells increases mostly due to the infiltration of microglia and macrophages and the massive proliferation of microglia. The numbers of neurons are severely reduced, and perineuronal nets and myelin sheaths are disrupted. One month or more after ischemia, the number of astrocytes increases, due to their proliferation and migration from the penumbra. The lesion contracts and the glial scar is stabilized by the extracellular matrix produced by reactive astrocytes. *Penumbra:* Swelling and activation of astrocytes, NG2 glia and microglia are delayed in comparison with the core but occur within 24 hours after ischemic insult. One to seven days after ischemia, neurons are still visible but their numbers have declined due to apoptosis. Astrocytes, microglia, NG2-glia and oligodendrocytes intensively proliferate. Proliferating astrocytes and microglia create heterogenic groups, where distinct subtypes differ in gene expression and membrane properties. Astrocytes and microglia with pro-inflammatory phenotypes in the vicinity of the core contribute to the formation of glial scar. Those with anti-inflammatory phenotypes in the outer parts of the penumbra with less severe hypoperfusion begin to produce growth factors and cytokines contributing to tissue regeneration. One month or more after ischemia, the numbers of glial cells are moderated, and depending on the duration and severity of the hypoperfusion, neurodegenerative or regenerative processes are activated. *Remote areas:* The first changes in the remote areas can be observed not earlier than 24 hours after ischemic insult but typically several days, weeks or even months following it. Depending on the brain region, changes in the cell structure and numbers are subtle or there are none. A slight decline in the numbers of neurons and a moderate increase in the numbers of microglia or astrocytes can be observed. However, morphological changes typical for their reactive states are mostly missing, even though the cells express markers of activation. More distinct alterations can be observed in the cellular functions, gene and protein expression profiles or production of cytokines. Induced delayed neuroinflammation may evoke neurodegenerative processes and amyloid deposits. For more details see the text.

Ischemia-induced changes in the core, penumbra and remote areas in time are described in more details in the chapters below and summarized in Figure 1.

### 3.1 Regional variability in zone definitions in rodent models of focal ischemia

The common regions of the necrotic core in rodent models of focal ischemia (see Glossary) are well known. Phototrombotic model of MCA blockage causes reproducible infarcts involving the parietal cortex in all its layers (Dihne et al., 2002; Reichmann et al., 2002; Schroeter et al., 2002; Haupt et al., 2007; Karetko-Sysa et al., 2011); other special types of phototrombotic models may cause necrosis for example in the caudoputamen (Kuroiwa et al., 2009). The striatum and cortex are consistently affected by the MCA occlusion (MCAO) in its proximal section using the Longa method (Tanaka et al., 2001; van Groen et al., 2005; Melani et al., 2006; Justicia et al., 2008; Bona et al., 2019) although the size may vary (Arlicot et al., 2010). On the other hand, the outcome of the proximal MCAO according to Koizumi, seems to be dependent on the duration of blood flow cessation. Short-term MCAO lasting 30 min leads to necrosis bound to the striatum (Arvidsson et al., 2001; Kronenberg et al., 2012; Prinz et al., 2015) longer occlusions (45–120 min) begin to involve the parietal cortex (Arvidsson et al., 2001; Bacigaluppi et al., 2009; Garbuzova-Davis et al., 2014; Cai et al., 2017; Gaire et al., 2019), and a 3-h occlusion spreads even further to the globus pallidus (Dihne and Block, 2001). The permanent version of the Koizumi method creates infarctions spanning over the striatum and almost the entire ipsilateral cortex (Takasawa K. et al., 2002; Ni et al., 2020). Distal MCAO produces reliable infarcts restricted to the somatosensory cortex (Hobohm et al., 2005; Ling et al., 2009; Cao et al., 2021; Ip et al., 2021). Researchers tend to get very similar results concerning the area of the ischemic core, yet for example, Popp et al. (2009) assume, that the core created after 120 min of proximal MCAO, also involves the amygdala and hypothalamus on top of the striatum and cortex, and that the penumbra spans into the hippocampus, thalamus and part of the hypothalamus (Popp et al., 2009). The thalamus, which has been extensively studied for its remote effects is, in the vast majority of publications, described as a primarily nonaffected structure (Dihne et al., 2002; Justicia et al., 2008), just like the hippocampus (Uchida et al., 2010). The discrepancies in the assignation of specific brain regions to either ischemic core, penumbra or remote areas may stem from the various methods used for their definitions. Thus, some studies rely on the definition depending on the acute reductions of blood flow (Sakoh et al., 2001; Yu et al., 2016), changes in the regional glucose metabolism (Szilagyi et al., 2012), the combination of hypoperfusion and the damage to the dendritic structure (Li and Murphy, 2008) or deviant signal intensity via MRI (Buffon et al., 2005).

## 4 The time course of stroke-induced changes in the core and the penumbra

### 4.1 Acute and sub-acute post-stroke reaction

The severely hypoperfused core is the location of the first reaction to ischemia. The first tissue reaction to the oxygen deficit is a substantial decrease in number of NG2-glia (Lee et al., 2003) and swelling of oligodendrocytes (Hernandez et al., 2021). Within the first 3 h post-ischemia, neurons with hyperchromatic nuclei can be observed (Yang et al., 2013). Four to 6 h following ischemia, the endothelial cells of the local capillaries become activated and the compromised integrity of the BBB results in edema. Hematoxylin-eosin staining can reveal red neurons indicating their damage (Hirouchi et al., 2007) and a general loss of cells. Barely any astrocytes can be seen (Nowicka et al., 2008; Aleithe et al., 2019) and those that remain are the source of the monocyte chemoattractant protein-1 (MCP-1) at 12 h after ischemia (Che et al., 2001). One day post-ischemia, macrophages/microglia are recruited into the core and their somas become larger, resembling an amoeboid shape. The tissue loses its clear structure, there is almost no immunostaining signal for neurons and the loss of *Wisteria floribunda* (WFA) staining with preserved proteoglycans indicates the partial decomposition of perineuronal nets (PNNs) (Hobohm et al., 2005). Axons begin to disappear (Khodanovich et al., 2018). The activated microglia and astrocytes also appear in the penumbra, and the astrocytes begin to form a wide layer around the ischemic core (Mabuchi et al., 2000; Melani et al., 2006; Nowicka et al., 2008). Glia are one of the main secretors of the ECM deposited in the lesion and the accumulated ECM macromolecules prevent axonal outgrowth in the area void of neurons (Dzyubenko et al., 2018). Both the NG2-glia (Tanaka et al., 2001) and astrocytes (Melani et al., 2006) are swollen, but the cell numbers do not differ. Although the number of neurons in the penumbra declines (Melani et al., 2006), the staining intensity for neuronal nuclear protein (NeuN) and microtubule-associated protein (MAP2), markers of mature neurons, remains constant (Cao et al., 2021). In contrast to the core, the PNNs in the penumbra are intact.

### 4.2 Early chronic post-stroke reaction

Massive gliosis spreads in the core on the third day after ischemia, with microglia proliferating to a large extent (Che et al., 2001); an increase in the number of glial cells can also be observed in the penumbra (Gaire et al., 2019). The release of the MCP-1 for the attraction of immune cells is intensified in the core and its spreading into the surroundings can also be observed (Che et al., 2001). Myelin sheaths in the core are damaged, the affected axons inside them separate from each other and vacuolization can be detected (Khodanovich et al., 2018). In the penumbra, neurons are still visible, in contrast to the core (Cao et al., 2021) and neural stem cells (NSCs) begin to appear in both structures (Shin et al., 2013). The MCP-1 expression dissappears from the core 5 days following ischemia, but still dwells in its outer rim (Che et al., 2001). One week after focal ischemia, the core still displays a minimum of neuronal cells and necrotic debris occupies the void (Schroeter et al., 2002). The phagocytic activity peaks at this time (Toth et al., 2016) as the core is packed with microglia (Michalski et al., 2017). NG2-glia are absent in the core, unlike in the penumbra, where they grow in size and numbers (Tanaka et al., 2001), presumably due to migration from the subventricular zone (SVZ) (Hernandez et al., 2021). Some of them acquire astrocyte-like phenotype and contribute to the formation of the glial scar (Valny et al., 2018). The structure overflows with astrocytes, microglia and oligodendrocytes (Mabuchi et al., 2000), and the glial scar becomes thinner as the astrocytes align more closely to each other (Hobohm et al., 2005; Nowicka et al., 2008; Cao et al., 2021). Ischemic injury evokes a reduction of NeuN^+^ neurons and a complete loss of MAP2^+^ neurons in the infarct core. In peri-infarct areas, the number of NeuN^+^ neurons is also decreased, but MAP2^+^ neurons are located around the infarct border (Cao et al., 2021). Hobohm et al. (2005) observed an appearance of aggrecan expression in reactive astrocytes 7 days after MCAO; the glial scar is already clearly visible at this time (Hobohm et al., 2005). The intensity of microglial Iba-1 staining reaches its peak in the core 2 weeks post-ischemia (Reitmeir et al., 2011). Myelination of the tissue remains low (Khodanovich et al., 2018) with a decline observed in the penumbra (Tanaka et al., 2016).

### 4.3 Delayed chronic post-stroke reaction

One month after ischemia, some axons are entirely demyelinated which negatively affects the interneuronal transport (Garbuzova-Davis et al., 2014). The loss of neurons is even more profound (Cao et al., 2021). The lesion contracts and contains a lot of swelling astrocytes, whose endfeet at the tip of the degenerating processes are detached from the capillary within the neurovascular unit (Nowicka et al., 2008). Microglia can be observed phagocytising around blood vessels (Garbuzova-Davis et al., 2014). The core is surrounded by a layer of polarized astrocytes with their processes extended toward the lesion (Nowicka et al., 2008). The immense numbers of glia in the penumbra are now moderated (van Groen et al., 2005). It takes a few more weeks for reactive microglia to diminish in the core, while the astrocytic scar becomes even more distinct (Reitmeir et al., 2011; Cao et al., 2021). Even 4 months after ischemia, some degenerated neurons may still be seen in the core and the total number of neurons is substantially attenuated (Cao et al., 2021).

## 5 The post-ischemic response in specific structures of the remote areas

Focal ischemic injury does not impact all remote regions in the same way. There may be various explanations for this heterogeneity, for instance, the existence of anatomical connections between the areas of the primary and the secondary lesion (Chen et al., 2014), unique vasculature (El Amki et al., 2015), the high density of receptors that mediate damage or recovery (Arvidsson et al., 2001; Onufriev et al., 2021).

### 5.1 Ipsilateral cortex

The cortex is typically the site of the ischemic core in the focal ischemia. However, only a part of the cortex can be truly necrotic, the rest may seem intact at first glance, especially during the acute phase. Apoptosis of **neurons** does not seem to occur in the remote cortex (Karetko-Sysa et al., 2011), yet this does not mean that cells are not impacted. For example, some researchers observed neurodegeneration in the remote ipsilateral cortex (Chen et al., 2014; Bona et al., 2019), although others claim no such damage was seen in their samples (Melani et al., 2006; Karetko-Sysa et al., 2011). It can be hypothesized that these different results might be attributed to the different time of post-stroke tissue evaluation (Minassian et al., 2019) or the type of an ischemic model (permanent versus reversible MCAO), where reperfusion can lead to more severe injury by the rapid burst release of reactive oxygen species (ROS) (Peters, 2006). Other neuron-associated changes, such as degeneration of axial dendrites with vacuolization and partial loss of synapses, were observed 7 days after photothrombosis, with a progressive deterioration in the following weeks (Lee et al., 2020). The increase in protein levels of growth associated protein-43 (GAP-43), a marker of axonal growth cones, indicates parallel damage and regeneration in the peri-ischemic cortex (Chen et al., 2014).

It was detected that the quantity and morphology of **astrocytes** in the ipsilateral medial frontal and cingulate cortex were constant 24 h after permanent MCAO (pMCAO) (Melani et al., 2006). Later stages are characterized by an elevation in GFAP expression, especially 4 and 7 days after ischemia, implying hypertrophy and increased proliferation of astrocytes (Nowicka et al., 2008). Ischemia ignites a wave of spreading depression in the astrocytic syncytium and the effects can spread into remote areas (Haupt et al., 2007). The number of cells expressing mRNA of connexin 43 (Cx43), the main component of astrocytic gap junctions (Liang et al., 2020), was reduced on day 1 following ischemia, slightly elevated on day 3, and substantially increased on day 7 in comparison to the contralateral (control) cortex. Subsequently, the amount of Cx43 mRNA-positive cells returned to levels comparable to the control 2 weeks after the ischemic injury (Haupt et al., 2007). These results indicate the temporary alterations in the intercellular connections within the astrocytic syncytium, that may affect the spread of calcium waves and injury propagation.

The remote cortex is a location where an inflammatory response can also be triggered, as was confirmed by the presence of heat shock proteins (Popp et al., 2009) and **microglia**. Microglial numbers were found to be highly elevated 1 and 3 days after proximal transient MCAO (tMCAO) (Gaire et al., 2019), just like in the core and penumbra, nevertheless, a closer look at the morphomolecular cell characteristics may show distinct reactions in each zone. It was proposed, that an intermediate state between resting and fully activated microglia exists - these cells have thin ramified processes similar to resting microglia, yet they expressed a marker of microglia activation, the purinergic receptor P2X7 (Melani et al., 2006; Monif et al., 2009).

In contrast to microglia, the **NG2-glia** seem to remain quiescent during ischemia in the remote areas, while they swell in the penumbra (Tanaka et al., 2001). To the best of our knowledge, no studies have investigated oligodendrocytes in the cortex beyond the penumbra so far.

The cortical **ECM** partially disappears upon ischemia. WFA staining revealed a decreased density of PNNs already 4 h after MCAO with an even more pronounced decline occurring 24 h after ischemia and restoration in the following week. The brevican immunoreactivity was also transiently attenuated 1 day after ischemia. This decline in ECM levels may be explained by enzymatic digestion (overproduction of MMPs and/or hyaluronidase) or reduced production of ECM by inhibitory neurons. On the other hand, ischemia did not affect the expression of aggrecan (Karetko-Sysa et al., 2011) or neurocan (Deguchi et al., 2005). One of the explanations for this diversity may be the contradicting actions of activated astrocytes and microglia, which produce both molecules of the ECM and the enzymes responsible for its degradation. Moreover, microglia may remove the macromolecules from the ECS by phagocytation (Dzyubenko et al., 2018; Raffaele and Fumagalli, 2022).

### 5.2 Hippocampus

The hippocampus is traditionally regarded as highly susceptible to global ischemia (Nikonenko et al., 2009; Baron et al., 2014), however, even focal ischemic injury can have a profound impact on it. The hippocampal damage in the ipsilateral hemisphere after focal ischemia is manifested, for instance, by the massive increase in apoptotic cells 2 weeks after tMCAO (Yang et al., 2019).

The first signs of **neuronal** degeneration can already be seen 12 days after MCAO in the cornu ammonis (CA) (Butler et al., 2002). Significantly reduced numbers of neurons can already be detected after 3 days (Uchida et al., 2010) and persist as long as 12 months after ischemia (Ouyang et al., 2020). A study by Park et al. (2011) was focused on the specific subsets of neurons in the CA1 and CA3 regions. They observed a decline in the numbers of cholinergic, NO-positive (NO^+^) and nitric oxide synthase-positive (NOS^+^) neurons, which were associated with impaired learning and memory in experimental animals (Park et al., 2011). In contrast, the study of Uchida et al. (2010) revealed increased levels of neuronal and inducible NOS (Uchida et al., 2010). Furthermore, they detected increased amounts of superoxide dismutase, which indicates augmented antioxidant activity.

However, other studies did not find any changes in neuronal numbers in the hippocampus, possibly due to only a brief interruption of the oxygen supply and examination of the histological results after 1 month, when the tissue might have undergone partial regeneration (Zhou et al., 2013; Brait et al., 2021). To compensate for the neuronal loss, the expression of doublecortin-expressing stem cells is increased in the subgranular zone (SGZ) of the dentate gyrus (Klein et al., 2016), which is one of the regions with preserved neurogenesis in adult life. The number of proliferating cells increases on the first day after proximal pMCAO, and reach their peak 4 days after ischemia. Around half of the progenitor cells begin to differentiate into neurons, whereas the other half develop into astrocytes (Takasawa K. et al., 2002).

The reaction of **astrocytes** to remote ischemia in the hippocampus are consistent: the GFAP levels and the numbers of GFAP-positive (GFAP^+^) cells increase shortly after the stroke (Haupt et al., 2007; Nowicka et al., 2008; Uchida et al., 2010) and remain elevated for several months (Ouyang et al., 2020; Brait et al., 2021). Butler et al. (2002) linked the augmentation of astrocyte activation with the appearance of degenerating pyramidal neurons (Butler et al., 2002), which likely occurred as a result of the astrocytic release of neurotoxic molecules (Phatnani and Maniatis, 2015). The time course of Cx43-positive cell numbers in the CA1 region differs from the one in the cortex: the boost starts from the first day and the rise is even stronger 1 week after ischemia (Haupt et al., 2007).

Experiments with proximal MCAO in male rodents led to substantial increases in Iba-1 protein levels and numbers of Iba-1^+^ **microglia** (Uchida et al., 2010; Brait et al., 2021). However, MCA photothrombosis in female mice did not impact the hippocampus, as this structure was only surrounded by activated microglia (Schroeter et al., 2002). These varying results may be attributed to the use of animals of different sexes as female mice are less susceptible to macrophage infiltration than males (Xiong et al., 2015). Alternatively, the use of the photothrombotic method, which creates precisely defined trauma may have prevented excessive spreading of the injury to the hippocampus (Clark et al., 2019).

Uchida et al. (2010) examined the fate of **oligodendrocytes** after ischemia and found a gradual decrease in their numbers with significant alterations in the third and seventh day after ischemia (Uchida et al., 2010).

Studies regarding the hippocampal **ECM** detected decomposed PNNs (Hartig et al., 2017) and attenuated immunostaining for type IV collagen, which could be explained by the vast expression of MMP-9 (Yang et al., 2019). The volume of the whole structure remained stable for the first 12 weeks after ischemia, followed by a minor yet significant enlargement (Brait et al., 2021).

### 5.3 Thalamus

The thalamus is the most explored structure in the subject of secondary post-ischemic damage. The intense focus on this region may be due to the existence of the physical axonal connection with the primary site of the insult, which can explain the diaschisis (Ouyang et al., 2020). There is already vast evidence showing that the thalamus is indeed impacted by ischemia in distant regions.

Hartig et al. (2016) found shrunken and fragmented GABAergic **neurons** from 1 day after ischemia in the reticular thalamic nucleus (Hartig et al., 2016). In contrast, Loos et al. (2003) did not see any abnormal morphology nor any decline in the number of neurons at that time (Loos et al., 2003). The discrepancy may be explained by the longer exposure to ischemia (pMCAO vs. proximal tMCAO) in the case of Hartig’s study or the use of different markers for neurons; parvalbumin expression can be found only in a subset of nerve cells, whereas NeuN is considered a pan-neuronal marker (Gusel’nikova and Korzhevskiy, 2015; Hartig et al., 2016). Most of the studies report the first changes occurring in the thalamus 1 week after focal ischemia, showing aberrant intercellular content (Dihne et al., 2002) and neuronal loss irrespective of the animal model used (Hirouchi et al., 2007; Wang et al., 2007; Ling et al., 2009; Chen et al., 2014; Ladwig et al., 2019; Xu W. et al., 2020). The levels of autophagy-related proteins Beclin 1 and MAP1LC3 (microtubule-associated protein 1A/1B-light chain 3) significantly rise (Xu W. et al., 2020). However, the number of neuronal cells remains reduced for months (Justicia et al., 2008).

Unlike in the ischemic core, **astrocytes** in the thalamus do not undergo any changes the first day after ischemia (Loos et al., 2003). The first signs of astrogliosis may be detected the third day after MCAO, which evolves fully 1 or 2 weeks after ischemia (Loos et al., 2003; Cao et al., 2021). The changes in activated astrocytes included upregulated GFAP expression (Loos et al., 2003; Hobohm et al., 2005), increased numbers (Dihne et al., 2002; Hirouchi et al., 2007; Xu W. et al., 2020) and cell swelling (Loos et al., 2003). Moreover, several studies even reported the formation of astrocytic scar (van Groen et al., 2005; Justicia et al., 2008). In contrast, one research group did not find any alterations in GFAP expression from 4 to 60 days after ischemia (Nowicka et al., 2008). Interestingly, Ling et al. (2009) reported the appearance of NSCs with morphological features of astrocytes, which grow in numbers the second week after ischemia and stretch first along the WM fibers and later accumulate in the ventroposterior thalamic nucleus (VPN) (Ling et al., 2009).

Similarly to the cortex and hippocampus, it is possible to observe activated **microglia** in the thalamus around the seventh day after ischemia, first with the hyper-ramified and later swollen shape (Dihne et al., 2002; Schroeter et al., 2002; Hobohm et al., 2005; Hirouchi et al., 2007; Wang et al., 2007; Ling et al., 2009; Klein et al., 2016; Ladwig et al., 2019; Xu W. et al., 2020). Of note, microglia-related genes are strongly upregulated in the ipsilateral thalamus a few days before the cell activation and the microgliosis persists for several months (Justicia et al., 2008; Cao et al., 2021). Chronic microglia activation is associated with the development of neurodegenerative diseases and can be an important link between stroke and post-ischemic Alzheimer’s or Parkinson’s disease (Lull and Block, 2010). Additionally, Smirkin et al. (2010) identified a subpopulation of microglia staining positively for Iba-1 and NG2, which is a distinct glia lineage with possibly neuroprotective properties (Smirkin et al., 2010). Interestingly, Ladwig et al. (2019) noticed an inverse proportion between the number of neurons and microglia: the more microglia, the fewer neurons (Ladwig et al., 2019). It was also observed that as soon as activated microglia appear in the thalamic tissue, the neurons begin to degenerate (Cao et al., 2021). Microglia were observed concentrated around amyloid β precursor protein (APP) deposits (Justicia et al., 2008). APP deposits, as well as amyloid β (Aβ) and Aβ plaques, can appear in the thalamus within weeks after ischemic insult and can persist for months (van Groen et al., 2005; Wang et al., 2007; Justicia et al., 2008; Lipsanen et al., 2011). The microglia can also collect iron, which may be accumulated in the APP deposits (Justicia et al., 2008). Moreover, the same study revealed increased transcription of heme oxygenase-1 (HO-1), an indicator of oxidative stress. All the processes imply the development of the post-ischemic tissue toward neurodegenerative dementia (Justicia et al., 2008). However, it is not possible to claim that solely microglia trigger neurodegeneration, although they substantially contribute to this process [for review, see (Harry, 2021)]. Clinical studies on post-ischemic secondary injury are rare, therefore we cannot draw any conclusions from them yet.

Another noteworthy event in the thalamus is the damage of the **ECM molecules**. WFA staining is strongly reduced from the PNNs the first day after ischemia; this effect is even more pronounced in aged mice (Hartig et al., 2016). The expression of other constituents of PNNs, such as aggrecan and neurocan, and generally of the chondroitin sulfate proteoglycans (CSPGs), is downregulated (Hobohm et al., 2005; Hartig et al., 2016).

Degenerating tracts of **white matter** between the thalamus and the cortex and atrophy of the entire structure were detected after stroke (Reichmann et al., 2002; Arlicot et al., 2010). In contrast to the ipsilateral cortex, the thalamic levels of GAP-43 and synapsin (the marker of synaptic vesicles) were diminished (Wang et al., 2007; Chen et al., 2014). This may suggest that synaptic regeneration is delayed in the thalamus. Alternatively, there are homeostatic control mechanisms counteracting the pathological direction of the thalamic tissue. For instance, cell proliferation begins no later than 1 week after ischemia and the intensity further increases in the second week (Ling et al., 2009). Some nestin and GFAP^+^ cells were detected along the corticothalamic fibers and in the VPN. These cells can later differentiate either into astrocytes or neurons (Witusik et al., 2008).

Tissue regeneration may be manifested in **angiogenesis**, i.e., the growth of new blood vessels importing oxygen and nutrients (Hatakeyama et al., 2020). Ischemia has been found to increase vascular density (Yanev et al., 2017), and capillary thickness and to trigger the proliferation of endothelial cells in the ipsilateral thalamus (Ling et al., 2009). The process is presumably stimulated by the release of angiogenic cytokines such as VEGF, MMPs, angiopoietins or basic fibroblast growth factor (BFGF) from the penumbral cells (Fang et al., 2023). Consequently, the volume of the thalamic blood flow increases (Yanev et al., 2017).

### 5.4 Substantia nigra

Although the substantia nigra (SN) is situated farther away from the common areas of the ischemic core, the secondary damage is evident. This might be caused by the disturbed interregional fiber connections (Hirouchi et al., 2007; Prinz et al., 2015), such as the nigrostriatal tract (Sonne et al., 2023). SN is the most explored brainstem structure for its crucial role in motor function, which frequently deteriorates after stroke (Prinz et al., 2015).

**Neurons** in SN are the first type of cells to respond to remote ischemia. The use of an electron microscope allowed for the observation condensed neuronal chromatin on the first day and degraded endoplasmic reticulum the second day after ischemia (Zhao et al., 2002). These details were not detected by other research groups, which reported healthy-looking neurons during the first week (Dihne and Block, 2001; Loos et al., 2003). Four days after ischemic insult, vacuolation of neurons occurs and their numbers start to decline. In cells with condensed cytoplasm, the plasmalemma later disintegrates (Zhao et al., 2002) and phagocytes remove the dead neurons (Dihne and Block, 2001). The cell loss is extensive, with a 52% decrease in neuronal numbers observed. This results in atrophy of the entire structure that can be detected 7 days after ischemia, and even more distinct shrinkage was evident 2 weeks following MCAO (Dihne and Block, 2001). In addition, the number of dopaminergic neurons, which comprise an important subpopulation of neurons in the SN, were also found to diminish the first week after ischemia (Huh et al., 2003; Prinz et al., 2015) and this decline was confirmed even several months following MCAO (Kronenberg et al., 2012). Nerve cells may transiently participate in the production of anti- or pro-inflammatory cytokines (Doll et al., 2014). In the remote SN, the intracellular presence of TNF-α (Loos et al., 2003) and IL-6 (Dihne and Block, 2001) were detected.

The **microglia** in SN become activated within the first week and their numbers increase (Prinz et al., 2015). Their shape becomes more hypertrophic, but transition into an ameboid shape was never observed (Dihne and Block, 2001; Huh et al., 2003). Similarly to the cortex, microglia in the SN are transformed into an intermediate semi-activated state. The duration of microglia activation seems to be somewhat shorter than in other regions as a maximum of 2 months was reported (Huh et al., 2003).

The changes observed in microglia are essentially very similar to those of the **astrocytes**. Astrocytes gradually swell (Loos et al., 2003), proliferate (Hirouchi et al., 2007) and their GFAP expression is upregulated (Dihne and Block, 2001), yet the intracellular space appears non-aberrant (Zhao et al., 2002).

In contrast, **oligodendrocytes** stay intact for several weeks after the insult (Zhao et al., 2002). Nevertheless, the effect of ischemia is potent enough to trigger angiogenesis and increase the perfusion of the SN (Yanev et al., 2017). No reports addressing alterations of the ECM in this region have yet been published.

### 5.5 White matter

The state of WM can be reflected by the post-stroke behavioral outcome as was reported by a clinical study, where a correlation between a long-term cognitive decline and a low integrity of exo-focal WM was observed (Schaapsmeerders et al., 2016). A comparable situation was seen in an animal study, where proximal tMCAO in aged mice caused severe **demyelination** of the corpus callosum (CC) 8 weeks after ischemia, which was associated with poor results in the corner and cylinder tests (Cai et al., 2017). Even more detailed results were shown by Wan et al. (2022), who reported that distal MCAO in young mice led to a significant decrease in the levels of myelin basic protein (MBP), myelin-associated glycoprotein (MAG), and neurofilament 200 (NF200) in the CC 7 days after ischemic insult. In addition, myelin density was diminished, the myelin integrity weakened, and its thickness was reduced by approximately one-half; the percentage of myelinated axons attenuated, and the diameter of myelin sheaths was reduced. The authors suggested that **astrocytes** may be partially responsible for the demyelination by their phagocytosis of ischemia-damaged myelin debris. They detected an increase in levels of GFAP protein and a reduction in the amount of an anti-inflammatory marker S100A10 (S100 calcium-binding protein A10). Furthermore, they detected an augmentation in levels of pro-inflammatory protein C3d (Complement component 3d), and an increased amount of lipocalin-2 protein, which is released by activated astrocytes and may be responsible for myelin degradation. All these astrocyte-related changes occurred at the same time as the WM destruction (Wan et al., 2022). However, not all animal studies concluded that ischemia must necessarily harm the WM. For example, one study found that the density of myelin fibers in the CC was unaffected 4 weeks after transient ischemia (Zhou et al., 2013). Reitmeir et al. (2011) obtained similar results in corticospinal tract fibers 52 days after ischemia (Reitmeir et al., 2011). Similarly, another study focusing on exo-focal changes in the thalamus and examining the internal capsule between the ischemic cortex and the thalamus did not find any pathological abnormalities (Dihne et al., 2002). It is difficult to establish a pattern which would explain these contradictory results. One may argue that senescence is a great contributor, as the extent of WM injury was found to be age-dependent (Rosenzweig and Carmichael, 2013). For example, one retrospective study (Yu S. et al., 2018), conducted on children with a history of perinatal stroke, revealed significantly lower myelinization in the contralateral hemispheres when compared to healthy controls, and even more profound loss of myelin in the ipsilateral remote areas. Other determinants may be the experimental species and the type of focal ischemia model.

As for cellular appearance in the fiber tracts, nestin-positive cells with astrocytic phenotype were detected in subcortical fiber tracts the first week after distal MCAO, before they spread into the adjacent thalamus (Ling et al., 2009). Interestingly, ischemic injury evokes the upregulation of **hyaluronan** (HA) throughout the ipsilateral CC and the peri-infarct area and accumulation of HA within the glial scar surrounding the lesion. Ischemia also induces the upregulation of HA synthase 2 (HAS-2) and hyaluronidase Hyal2 production, as well as the expression of Rhamm (hyaluronan receptor) in astrocytes (Lindwall et al., 2013). Greda and Nowicka (2021) investigated HA metabolism in the ischemic brain and found that all 3 synthases (HAS1-3) and 2 hyaluronidases (Hyal1, 2) were affected. The authors also showed that inhibition of hyaluronidase improves behavioral outcomes after stroke (Greda and Nowicka, 2021). Additionally, cell culture experiments suggest that parameters, such as changes in HA concentration, can affect astrocytic reactivity and/or contribute to the inflammatory response of astrocytes (Jimenez-Vergara et al., 2020). It is possible to spot activated hypertrophic **microglia** in the WM (Schroeter et al., 2002), although they never acquire the amoeboid shape observed in other remote areas (Reichmann et al., 2002). Unsurprisingly, Cai et al. (2017) identified impaired **oligodendrocytes** which can be associated with the damaged fibers they ensheath (Cai et al., 2017). The consequence of disturbed WM is reduced anisotropy along the ipsilateral pyramidal tract in the section between the thalamus and the midbrain in patients 6 months after stroke (Buffon et al., 2005).

### 5.6 Other structures of the ipsilateral hemisphere

In this chapter, we will describe the post-ischemic changes occurring in the less studied remote brain regions such as the *cerebellum*, *pons*, *midbrain*, *ventral tegmental area*, *amygdala* and *basal ganglia*.

#### 5.6.1 The cerebellum

The diffusion of water, neurotransmitters, ions or gases may be hindered as a result of ischemia. One hour after MCAO, Yang et al. (2013) described a reduction of apparent diffusion coefficient of water (ADCw) by approximately 58 % in the ischemic core in the left hemisphere, by 24 % in the right and by 20 % in the left cerebellar hemisphere (Yang et al., 2013). It may seem paradoxical, that the greater change occurred in the contralateral cerebellar hemisphere rather than in the ipsilateral one, which is located closer to the core. The rationale for this may be the crossed cerebellar diaschisis, a term used for the reduction of blood flow and metabolism in the contralateral cerebellum after remote supratentorial infarction (Sobesky et al., 2005; Madai et al., 2011). The exo-focal injury occurs on the opposite side to the core due to the existence of the cortical-pontocerebellar tract (Lin et al., 2009), which is the main source of input from the cerebrum to the cerebellum and which crosses to the other side at the level of the pons (Kamali et al., 2010). The phenomenon of diaschisis was associated with clinical outcomes and was considered a potential indicator of recovery development (Lin et al., 2009). Similarly to the study mentioned above, Ma et al. (2022) found significant decreases in cerebellar diffusivity. However, their results differ in the cellular reaction to diaschisis (Ma et al., 2022). While Yang et al. (2013) described healthy-looking **neurons** with preserved numbers, Ma et al. (2022) noticed a high number of apoptotic cells, especially 24 h after ischemia (Yang et al., 2013; Ma et al., 2022). Another publication reported a huge increase in the number of apoptotic cells in the contralateral cerebellum when compared to the ipsilateral part of the structure. Interestingly, in transcriptional gene analysis, they did not detect an upregulation of genes related to apoptosis, hypoxia or ROS, although the mRNA levels of HO-1 and Nrf-2 (nuclear factor erythroid 2-related factor 2), which are considered markers of oxidative stress, were increased. The only set of cerebellar genes enhanced by supratentorial ischemia were those involved in oxidative phosphorylation (Kidani et al., 2020). Concerning **microglial** reaction, PET experiments observed no rise in the binding of [^11^C](R)-PK11195 in either cerebellar hemispheres (Gerhard et al., 2005), suggesting an absence of activated microglia/macrophages.

#### 5.6.2 Other ipsilateral regions

The spread of microglia/macrophages from the core to the ipsilateral *pons* occurred in one patient 150 days after stroke. A similar clinical study found increased [^11^C](R)-PK11195 binding in the pons and midbrain in patients with damaged pyramidal tracts (Radlinska et al., 2009). The following investigations revealed persisting microglial activation 6 months after ischemia without a significant decrease and this cell activation was positively correlated to improved motor function (Thiel et al., 2010). Undisturbed motor function is also regulated by dopaminergic neurons. Their quantity was diminished in the ipsilateral ventral tegmental area several months after proximal tMCAO (Kronenberg et al., 2012).

If we focus on cellular changes in other brain regions, we may find evidence of astrogliosis in the *amygdala* triggered by cortical photothrombosis (Nowicka et al., 2008). However, neurons and astrocytes in the *ventral pallidum* and *olfactory tubercle* have unchanged morphology and density 1 day after ischemia (Melani et al., 2006). Demyelination and oligodendrocytic damage were also observed in the remote *striatum.* However, the striatal WM damage is balanced by angiogenesis, when the number and length of blood vessels were enhanced compared to sham animals (Cai et al., 2017).

### 5.7 Contralateral hemisphere

The search for any post-ischemic alterations in the contralateral hemisphere might seem futile. After all, the results obtained from the ipsilateral hemisphere are often compared to those in the contralateral one. Nevertheless, there is disagreement among various studies regarding the reliability of using the contralateral hemisphere as a control. There are numerous publications, which reported no changes in the contralateral hemisphere. For example, Sehara et al. (2006) did not observe any alteration in NeuN and GFAP expression, cell proliferation rates, and/or in numbers of cells positive for inducible NOS (Sehara et al., 2006). In the subacute post-ischemic period, no damaged nerve cells were detected in the contralateral hippocampus or SN either (Dihne and Block, 2001; Uchida et al., 2010). Another study found astrocytes and microglia with no signs of activation in the contralateral cortex, hippocampus or amygdala (Nowicka et al., 2008). In addition, Reitmeir et al. (2011) did not observe any Iba-1^+^ microglia or CD45^+^ leukocytes in the whole contralateral hemisphere (Reitmeir et al., 2011). Nevertheless, there are numerous examples of ischemia affecting structures beyond the brain midline. For example, some studies describing astrocyte activation, abnormal organelle morphology and changes in their end-feet in the contralateral cortex and striatum 7 days after focal ischemia have been published (Garbuzova-Davis et al., 2013, 2014). Moreover, the authors detected alterations in the neuronal chromatin content, attenuation of the nerve cells’ number, activated microglia, fewer myelin sheaths and diminished myelin staining, as well as in changes in the BBB integrity, which led to plasma leakage, resulting in extracellular edema (Garbuzova-Davis et al., 2013, 2014). Another study by Bona et al. (2019) reports a minor but significant decrease in the density of Iba-1^+^ cells in the contralateral cortex and striatum when compared to the baseline in contrast with the ipsilateral hemisphere, where these cells were packed. The authors suggested the possibility that microglia from the contralateral hemisphere migrated to the ischemic lesion. They also observed a decrease in early neuronal NO content in the contralateral regions homologic to the necrotic core, and an increase in the number of degenerating Fluoro-Jade-positive neurons from the first to the seventh day after ischemia (Bona et al., 2019). The post-ischemic response of regions with preserved adult neurogenesis was also investigated. Takasawa K. et al. (2002) reported an increase in proliferating bromodeoxyuridine (BrdU)-positive cells (BrdU^+^) in the SGZ of the contralateral hippocampus 7 days after pMCAO and a return to control levels after 14 days. Around 80 % of these cells were neuronal stem cells also positive for markers Musashi 1 and doublecortin and the rest expressed GFAP. Four weeks after ischemic insult, the majority of BrdU^+^ cells expressed both NeuN and MAP2, and 10 % of these proliferating cells colocalized with GFAP. Despite this, the cell proliferation remained unchanged in the SVZ (Takasawa K. et al., 2002). The ECM may undergo post-ischemic alterations since the density of WFA-positive PNNs in the contralateral cortex was decreased several hours after ischemia, and this reduction lasts for several months (Karetko-Sysa et al., 2011). In contrast, Deguchi et al. (2005) detected a slight increase in the levels of neurocan in the same region (Deguchi et al., 2005). Another study observed a substantial increase in the expression of CSPGs 3 days after ischemia and its moderate reduction 3 weeks later, yet a complete return to the control values was not detected (Liu et al., 2014).

## 6 Possible mechanisms of remote post-ischemic damage

So far, we have described the diverse changes occurring in remote areas following ischemia. However, the processes that enable the spread of the injury into distant places are still not fully elucidated. In this chapter, we will describe the possible mechanisms that could enable injury propagation into the other brain structures.

### 6.1 Damage to the white matter

Most of the studies explain the induction of the secondary injury by the interrupted communication between the core region and the remote structure. The disconnection may be caused by the physical destruction of the connecting fiber tracts (Reichmann et al., 2002) or by waves of spreading depression (Witte et al., 2000; Arvidsson et al., 2001; Haberg et al., 2009), which prevents the generation of action potentials in the affected tissue. The crossed cerebellar diaschisis is unanimously explained by the diminished excitatory activity from the site of injury to the contralateral cerebellum (Gold and Lauritzen, 2002; Lin et al., 2009; Poretti and Boltshauser, 2012; Takuwa et al., 2013). On the other hand, the effect of the ischemic core damage on the thalamus may be rather bidirectional: Ling et al. (2009) propose that anterograde degeneration is the underlying mechanism (Ling et al., 2009), just as in the cerebellum, while others believe that it may be caused by retrograde degeneration (Buffon et al., 2005; Wang et al., 2007; Arlicot et al., 2010). It was also speculated that debris from the deteriorated proximal nerve end is phagocytized by activated microglia, which then degrade the adjacent PNNs (Hobohm et al., 2005). In either case, the thalamic reaction certainly seems to result from damaged projections to the area from the ischemic core, as the changes in the first 2 weeks occurred only in the VPN that is structurally connected with the damaged cortex, while other nuclei remained intact (Wang et al., 2007). The SN is connected to the ischemia-affected striatum by GABAergic pathways. It was suggested, that in contrast to the cerebellum and thalamus, the SN is overwhelmed by the excitatory input since the inhibitory pathways from the damaged striatum are deafferented (Zhao et al., 2002). A different approach was used to elucidate the exo-focal injury in the hippocampus: a recording of local field potentials revealed disturbed sharp-wave associated ripples in the CA1 region and diminished theta-gamma coupling between the hippocampus and the lesioned sensorimotor cortex, which may manifest in post-stroke cognitive and memory deficits (Ip et al., 2021). In a study by Gold and Lauritzen (2002), functional ablation of the cortex (initiated by tetrodotoxin application or by triggering of spreading depression) was able to simulate the post-ischemic processes with a reduced spike activity of Purkinje cells in the contralateral cerebellum. Moreover, another common feature of focal ischemia and the artificially elicited cortical dysfunction was markedly reduced cerebellar blood flow (Gold and Lauritzen, 2002).

### 6.2 Hypoperfusion

Disturbances of regional blood perfusion, together with a decrease of neuronal metabolism as a consequence of attenuated input from ischemic areas, were suggested as another possible mechanism explaining the exo-focal changes (Lin et al., 2009; Agrawal et al., 2011). Indeed, hypoperfusion in regions beyond the penumbra was observed both in human (Kamouchi et al., 2004; Lin et al., 2009; Madai et al., 2011; Jeon et al., 2012) and in experimental animals (Martin et al., 2012; Takuwa et al., 2013; Kidani et al., 2020). Experiments using PET were also able to show reduced metabolic activity within several weeks after ischemic injury in the remote cerebellum (Joya et al., 2018) and cortex (Lee et al., 2020).

### 6.3 Compromised BBB integrity

The remote tissue is undernourished, and the endothelial cells stand in the first line to experience reduced nutrient supply via blood vessels. We have already detailed the damage to the BBB above. Capillaries in remote regions are delineated by degenerating endothelial cells and pericytes, with basal lamina detached from the blood vessel lumen and astrocytic end-feet (Garbuzova-Davis et al., 2013). Sun et al. (2017) suggested that the increased permeability of the endothelial layer in non-ischemic regions may be caused by augmented MMPs production and diminished levels of tight-junction proteins mediated by the hypoxia-inducible factor 1 (HIF-1) (Sun et al., 2017).

### 6.4 Edema

The breach in the BBB leads to fluid leakage into the tissue and the formation of vasogenic edema (Witte et al., 2000; Dihne et al., 2002). For instance, increased volume of the ipsilateral and later the contralateral hippocampus was observed within several hours after MCAO in rats. The edema is thought to spread gradually from the place of ischemia, which can explain the earlier swelling in the ipsilateral hemisphere (Izumi et al., 2002). It was hypothesized, that the edematic tissue in the intracranial cavity has no extra space to expand and starts to compress the adjacent capillaries (Bona et al., 2019). The blood pressure is therefore insufficient to nourish the tissues and mild local ischemia occurs. This deprivation of oxygen and glucose may lead to similar alterations that occur in the primarily affected tissues, although with smaller intensity. The tissue edema might be absorbed or in some regions not created at all, which leaves an unrestricted route to transport substances from the ischemic lesion to the rest of the brain via blood or cerebrospinal fluid (Witte et al., 2000).

### 6.5 Increased levels of pro-inflammatory compounds

The systemic levels of pro-inflammatory cytokines are elevated from several hours up to days after stroke (Doll et al., 2014). To the best of our knowledge, there is no study verifying/disproving the presence of increased unbound inflammatory substances in the capillaries of remote areas. However, immunostaining confirmed the expression of the IL-6 (Dihne and Block, 2001), TNF-α (Loos et al., 2003) and C3d (Wan et al., 2022) in cellular components. On the other hand, these compounds may not be transported to the remote regions but are created *in situ* as well.

### 6.6 Propagation through astrocytic syncytium

Another factor, which may contribute to the propagation of ischemic damage is the astrocytic syncytium, which aids in the transfer of ions and small molecules from the ischemic core. This can be an effective mechanism for dispersing the accumulated harmful substances from the core to its surroundings and thus attenuating the damage caused in the most endangered region. However, it also means, that the healthy tissue is introduced to the same noxious material, such as lactate (Rossi et al., 2007). It was suggested, that hemichannels open with delay after the onset of ischemia (Davidson et al., 2013; Kim et al., 2016); this could partially explain the slower post-ischemic reaction in remote areas. Moreover, astrocytes are able to produce waves of intracellular calcium and propagate thus the noxious activity via the syncytium over long distances. Activated astrocytes residing in remote areas can cause considerable harm on their own by the release of glutamate, reactive oxygen/nitrogen species or ATP (Verkhratsky, 2007).

### 6.7 Excessive microglial activation

The release of glutamate (Kaushal and Schlichter, 2008) and ATP was found to activate microglia (Takano et al., 2009) and consequently trigger a production of inflammatory cytokines and MMPs (Yenari et al., 2010). The phagocytes can start eliminating healthy cells and the cytokines and the activation of MMPs contribute to BBB damage, favoring the extravasation of more macrophages, which exacerbates the inflammatory reaction (Woodburn et al., 2021). A formerly intact tissue may thus gradually become truly damaged.

All these potential mechanisms of remote injury have been described in the penumbra; their role in spreading the tissue damage into distant regions needs to be explored by further studies.

## 7 Potential therapeutic targets

Knowledge of the processes occurring in the remote areas and what possibly drives them, gives us key information about the targets for therapy.

### 7.1 Attenuation of microglial activation

Excessive microglial activation needs to be modulated in order to prevent damage during the removal of dysfunctional elements (Jia et al., 2021). For example, knocking-down the lysophosphatidic acid receptor 1 (LPA1), one of the triggers of microglia activation, decreases the severity of neuropathic pain (Gaire et al., 2019). The study also showed that the *LPA1 antagonist AM095* decreased the number of Iba-1^+^ cells in the peri-ischemic cortex and promoted a slender ramified microglia morphology. Similar results were observed with the application of *osteopontin*, a glycoprotein expressed mainly in bone but also in microglia and macrophages, that was shown to reduce the number of microglia and GFAP^+^ cells in the ipsilateral thalamus (Mazzali et al., 2002). Osteopontin did not have any negative effect on neurons, on the contrary, it prevented neurodegeneration. The numbers of phagocytising cells and hypertrophic microglia were diminished after its application (Ladwig et al., 2019).

Microglial activation is commonly perceived as a phenomenon, which has to be controlled. Nevertheless, based on the observation of increased microglia reactiveness in the contralateral hemisphere together with an improved neurological score after antagonism of P2XP2Y receptors with *Reactive Blue 2*, it was suggested that the higher activity of microglia may accelerate the recovery process (Melani et al., 2006).

### 7.2 Stimulation of tissue regeneration

*FG loop* (FGL) is part of the neural cell adhesion molecule and was previously found to be an anti-inflammatory substance that promotes the migration of neural precursor cells. Regular subcutaneous injections of FGL to rats with MCAO significantly increased the number of cells positive for doublecortin in the ipsilateral SGZ 7 days after ischemic insult. FGL also supported the proliferation of NG2-glia in the remote WM and the thalamus as well as an increase in the number of Iba-1^+^ microglia in both the ipsi- and contralateral thalamus. However, FGL administration did not affect microglial polarization toward the M2 type in the thalamus (Klein et al., 2016). Cheng et al. (2019) conducted a consequent study, with experiments discovering the vessel-protective effects of *microRNA-195* (Cheng et al., 2019), which is a small set of nucleotides regulating gene expression in various diseases (Yu W. et al., 2018). Its administration was subsequently associated with an increased number of cells positive for GAP-43, which was highly expressed in neuronal growth cones.

### 7.3 Blockade of Nogo-A

Several studies also tested *an antibody against Nogo-A* (neurite outgrowth inhibitor-A), which suppresses the growth of axons. The hypothesis that the antibody could increase the number of pyramidal tract fibers in the pons was not confirmed, although the density of fibers increased in the cervical spine (Wiessner et al., 2003). The use of another *inhibitor against Nogo-A (NEP1-40)* prevented the ischemia-affected loss of GAP-43-positive axons in the VPN. It decreased the expression of APP in the second and fourth week after ischemia as well, however, the neurological score did not improve throughout the 4-week duration of the experiments (Wang et al., 2007). The third study tested NEP1-40 in the thalamus as well, and confirmed that the levels of Nogo-A were boosted in animals 7 days after MCAO. The inhibition of Nogo-A caused an increase in the number of intact neurons and a decrease in the number of GFAP^+^ and Iba-1^+^ cells compared to the vehicle group. The results also suggest that the RhoA/ROCK (Ras homolog gene family member A/Rho-associated protein kinase) pathway and excessive autophagy were limited after Nogo-A inhibition. The treated rats then showed better somatosensory function than untreated animals during the adhesive removal test (Xu W. et al., 2020). A comparison of the latter two studies shows that administration of a higher dose for a shorter time is more beneficial than the distribution of the drug intake over a longer period. Nevertheless, the question remains, whether the improved neurological state can be ascribed to recovery of the thalamus or the cortex.

### 7.4 Suppression of excitotoxicity and inflammation

Excitotoxicity may cause distress in remote areas as increased content of glutamate was confirmed shortly after ischemia in the contralateral hemisphere (Bona et al., 2019). Prinz et al. (2015) assessed *dizocilpine*, an antagonist of the N-methyl-D-aspartate (NMDA) receptor, and showed that the drug caused a considerable increase in the number of neurons in the ipsilateral SN but it did not have any effect on microglia (Prinz et al., 2015). The authors also tested *tacrolimus*, a known immunosuppressant utilized for the prevention of organ transplant rejection. However, the delayed administration of this anti-inflammatory agent did not provide any neuroprotection (Plosker and Foster, 2000; Prinz et al., 2015).

### 7.5 Removal of barriers limiting neuronal sprouting

The ECM in the form of PNNs stabilizes the synapses in the adult brain, but their presence impedes the neuroplasticity needed for post-injury regeneration. Moreover, ECM as a part of the glial scar prevents axonal ingrowth into the lesioned ischemic core. To promote after-ischemic regeneration, these restrictions should be eliminated, for example enzymatically. Chen et al. (2014) exploited the ability of *chondroitinase ABC* to cleave CSPGs by its direct pumping into the ischemic lesion. The number of neurons and the expression of GAP-43 and synapsin in the ipsilateral VPN was substantially higher in treated post-ischemic animals in comparison with untreated ones. The authors suggested that chondroitinase could not affect the VPN directly, as they did not detect any CSPG digestion in the thalamus and the molecule is too large to be conveyed into exo-focal regions. They thus assume that the enzyme had a local effect in the core and influenced the spread of neurodegeneration via the corticothalamic tracts (Chen et al., 2014).

### 7.6 Estrogen administration

Despite the higher lifetime risk of stroke in men, women often experience more severe strokes, increased stroke-related deaths, and elevated post-stroke functional deficits, especially following the menopause (Appelros et al., 2009). Ovarian hormones, acting via genomic and non-genomic receptors, significantly influence the neuroprotective role in acute conditions such as ischemic stroke, and traumatic spinal cord and brain injuries. They directly impact neuronal death pathways and modulate the immune system, with outcomes being dose-dependent and age-related (McEwen and Milner, 2017; Spychala et al., 2017; Kim et al., 2019). Investigations reveal that females demonstrate post-ischemic upregulation in TNFR1 (tumor necrosis factor receptor-1), IL-17, and natural killer cell signaling pathways, while males manifest increased expression in components of pathways linked to cell development, cellular migration, and pro-inflammatory reactions (Turtzo et al., 2011; Cabrera Zapata et al., 2022). Based on these findings, the potential therapeutic role for estrogen in stroke intervention was proposed (Sohrabji et al., 2019).

The influence of the estrogen cycle in female animals on stroke outcomes is a complex and multifaceted area of research. The function of estrogen, primarily *17β-estradiol*, extends beyond its reproductive role to impact pathological processes, particularly in the CNS (Rexrode et al., 2022; Zhang et al., 2023). Numerous studies highlight its protective role in brain injuries following ischemic stroke, involving the modulation of local and systemic immune responses post-stroke onset (Petrone et al., 2014; Patkar et al., 2022). After a stroke, 17β-estradiol demonstrates neuroprotective effects by moderating inflammation, activating macrophages, and releasing anti-inflammatory cytokines (IL-10 and TGF-β) (Akabori et al., 2010; Acosta-Martinez, 2020). According to Dang et al. (2011), a combination of 17β-estradiol with *progesterone* attenuates the expression of pro-inflammatory chemokines or IL-6 and induces the expression of VEGF. The authors also found decreased microglial activation (decreased Iba-1 or CD68) in the penumbra and decreased infarct size after treatment (Dang et al., 2011). Additionally, 17β-estradiol showed protective properties against lipopolysaccharide-induced microglial activation in the cortex, striatum, amygdala, thalamus, medial forebrain bundle and hippocampus (Suzuki et al., 2009).

However, findings regarding the neuroprotective effect of estrogens in stroke are not consistent. In tMCAO ischemia models, estradiol typically shows beneficial effects on stroke infarction. In contrast, in permanent ischemia models, some studies suggest that estrogen may contribute to an increase in infarct volume (Carswell et al., 2004; Bingham et al., 2005; Gordon et al., 2005). Acyclic middle-aged female rats, characterized by reproductive senescence, display significantly larger infarct volumes compared to young females. These findings align with the hypothesis that ovarian aging impairs stroke recovery. Notably, estradiol treatment, which decreases infarct volumes and reduces sensory-motor impairment in young females, paradoxically increases infarct volume in reproductive senescent females (Glendenning et al., 2008; Selvamani and Sohrabji, 2010). Choi et al. (2021) conducted a study revealing that the E/T (estradiol/testosterone) ratio is increased during acute stroke and predicts unfavorable early functional outcomes. They identified a significant association between the highest tertile of the E/T ratio and stroke. These results propose the E/T ratio as a potential independent biological marker for stroke, where the elevated value predicts early unfavorable functional outcomes (Choi et al., 2021).

### 7.7 Dietary intervention

The amount of *polyunsaturated fatty acids* (PUFAs) declines with age. Their intake was shown to contribute to the recovery of the ischemic brain in young animals. A diet enriched in ω3 PUFAs given long before and after tMCAO encouraged angiogenesis in the striatum beyond the penumbra. Moreover, it increased the quantity of the migrating NSCs, and differentiated neurons and attenuated the WM injury. All the results positively correlated with improved sensorimotor functions of the animals (Cai et al., 2017).

### 7.8 Plant-based drugs

Laboratories in East Asia have a long tradition of experiments with plant-based medicine (Chen et al., 2019). An extract from *black ginseng* is claimed to possess anti-inflammatory and antioxidant properties. Daily oral administration of the extract for 2 weeks compensated for the ischemic loss of cholinergic neurons, neurons expressing NOS and the nerve cells in general in the ipsilateral hippocampus. In the Morris water maze test, mice supplemented with black ginseng after MCAO were able to find the invisible platform sooner than the untreated animals. The protection of cholinergic corticohippocampal circuits due to black ginseng administration was proposed to retain spatial learning ability (Park et al., 2011). Similarly, *astragaloside*, a saponin from *Astragalus membranaceus*, is believed to suppress oxidative stress and inflammation. Administration of astragaloside IV caused the infarct size reduction and significantly boosted the number of NSCs in the dentate gyrus and nestin mRNA on days 3, 7 and 14 after ischemia. The drug also increased the content of BDNF in the hippocampus, and it was demonstrated that the above-mentioned effects are carried out via the BDNF-TrkB signaling pathway (Ni et al., 2020).

### 7.9 Off-label use of medicine

Experiments with medical drugs already used for other conditions spare the researchers the lengthy testing for safety and the financial exhaustion associated with clinical trials. *Fingolimod* is a drug approved for the treatment of multiple sclerosis and its mechanism of action is the modulation of sphingosine-1-phosphate receptor. A single i.p. injection shortly after MCAO attenuated the inflammatory reaction in remote areas by reducing the amount of ICAM-1 (intercellular adhesion molecule 1) expressed in blood vessels and of the number of Iba-1^+^ cells (Wei et al., 2011). A further logical step would be to test a common anti-inflammatory drug. Lipsanen et al. (2011) evaluated the effect of *ibuprofen* on gliosis in the ipsilateral thalamus of young male rats after MCAO. However, even a month-long daily administration of peroral ibuprofen did not decrease the activation of GFAP^+^ cells and the area containing microglia was even significantly extended (Lipsanen et al., 2011). On the contrary, an active form of *vitamin D_3_* was able to reduce the number of GFAP^+^ astrocytes in the remote cortex the second day after ischemia, if administered early after the ischemia onset, while later applications did not have any effect (Oermann et al., 2004). *Rosiglitazone* is a medicine used for lowering glucose blood levels (Deeks and Keam, 2007). It belongs to a group of PPAR-γ (peroxisome proliferator-activated receptor γ) agonists and its positive effects on brain infarction have been confirmed. Daily i.p. injections caused a moderate increase in the number of oligodendrocyte precursors and their proliferation in the SGZ of the dentate gyrus and diminished sensorimotor deficits (Han et al., 2015). Another approach was based on the idea that hypoxic stress triggers adaptive brain remodeling. *Deferoxamine* (DFX), a chelating agent used to treat iron or aluminum toxicity, is classified as a prolyl hydroxylase inhibitor, functioning as a neuroprotective agent through the facilitation of HIF-1 accumulation. HIF-1 serves as a pivotal transcription factor, critical for cellular and organismal adaptation to hypoxic conditions and is frequently found in ischemic tissue. The activation of HIF-1 initiates the expression of numerous targets, encompassing proteins that enhance angiogenesis, elevate glycolytic activity, and mitigate ROS production derived from mitochondria (Semenza, 2000; Freret et al., 2006). DFX enhances the expression of HIF-1 target genes, leading to a substantial reduction in stroke volumes (Siddiq et al., 2005; Baranova et al., 2007). The study of Freret et al. (2006) has shown that the application of DFX to male rats diminished the shrinkage of the thalamus, in contrast to the core areas, where it did not affect the volume of necrotic tissue after ischemic injury (Freret et al., 2006). However, certain *in vitro* studies have proposed that DFX induces neuroprotection independently of HIF-1 function (Siddiq et al., 2009; Niatsetskaya et al., 2010). The HIF-1 independent mechanisms is also suggested by results obtained in transgenic mice haploinsufficient for HIF-1 or with conditional loss of HIF-1 function in neurons and astrocytes, where the neuroprotective effect of DFX was preserved (Zhao and Rempe, 2011).

### 7.10 Biologic drugs and approaches

Neurons, astrocytes and endothelial cells express a receptor for *erythropoietin*, a growth factor contributing to axonal outgrowth in the CNS (Reitmeir et al., 2011). Erythropoietin administered into cerebral ventricles for the whole month following ischemia promoted the outgrowth of midline crossing fibers from the contralesional pyramidal tract at the level of the red nucleus and facial nucleus, but not those on the ipsilateral side. It attenuated the expression of IL-β, GFAP and the TGF-β in the parietal cortex of the contralateral hemisphere as well. The positive results of the study were explained by an increase in the expression of growth factors and other substances supporting brain plasticity such as BDNF or insulin-like growth factor (Reitmeir et al., 2011).

Another study testing the transplantation of *neurospheres containing NSCs* from the SVZ of healthy adult mice into animals after MCAO showed that the transplant administration significantly increased the thickness of the remote CC (Bacigaluppi et al., 2009). Another study used stem cells in the form of autologous mononuclear bone marrow cells that were administered intravenously. The transplant reduced axonal degeneration in the WM of the remote zone and the contralateral hemisphere and completely prevented lymphocytic infiltrations around capillaries. Additionally, the recruitment of microglia was attenuated in the contralateral hemisphere (Boltze et al., 2011).

Stem cells are not necessarily introduced into the brain artificially, but their production can also be triggered by an external method such as a *non-convulsive electrical stimulation* using ear-clip electrodes. Delayed stimulation of aged rats caused a massive increase in the number of newborn migrating neurons in the ipsilateral SGZ and SVZ 48 days after distal MCAO. The treatment also improved spatial memory and sensorimotor function of the forelimbs, without any effect on gross motor skills (Balseanu et al., 2020).

## 8 Future outlook, recommendations for further studies

In this review, we aimed to summarize the results of more than 20 years of research on the topic of post-ischemic changes in remote areas. Yet, there are still many gaps in the research that need to be filled.

### 8.1 Studying the unknown

Regarding the focus of the studies, a rather neuron-centric approach is still present. In order to see the whole picture, filling in the mosaic with information on all its components is necessary. There is still a lack of data on the roles of oligodendrocytes and NG2-glia in ischemic remote areas. Furthermore, since many researchers suggest that remote injury, especially in the thalamus (see chapter “The post-ischemic response in specific structures of the remote areas”), occurs due to the damage to cortical fiber tracts, future research should be focused on myelinization or the support of oligodendrocytes and axonal growth. Widening the narrow array of studies evaluating the components of ECM may also be beneficial, as ECM can have both positive and negative effects on the injured tissue. The ECM provides a protective coating around cells (Bozzelli et al., 2018), but it may also hamper tissue remodeling (Dzyubenko et al., 2018). Some of the brain structures should not be forgotten in the research, since their investigation may be relevant for post-stroke consequences. For instance, ischemic episodes are often followed by motor dysfunctions, which implies the involvement of the motor cortex (Li et al., 2016). The amygdala is poorly investigated, even though it has an important role in the development of depressive disorder, another common consequence of stroke (Wijeratne and Sales, 2021). Furthermore, the state of the hippocampus may reflect both depression and cognitive impairment that frequently appear in post-ischemic patients (Gulyaeva et al., 2021). Finally, the majority of explanations of the mechanisms of secondary damage given in the publications are speculations, and more experiments assessing their verity are needed. In particular, the roles of spreading depolarization and propagation via astrocytic syncytium are necessary to clarify.

### 8.2 Employing imaging-based approaches

Advanced imaging methods are a valuable tool for assessment of the ischemic injury propagation into the remote areas along pathways connecting different brain structures. These techniques play an important role in investigating brain connectivity and cognitive functions (Morita et al., 2016) and may allow the possibility to elucidate and quantify connectivity-driven cellular changes after stroke. Two key aspects are commonly explored: structural connectivity, which involves mapping the physical connections between brain regions (diffusion tensor imaging (DTI), tractography), and functional connectivity, which assesses the synchronization of neural activity between different brain regions (functional MRI, task-based functional MRI) (Meier et al., 2016). These methods are crucial for connectomic studies that explore how alterations in structural connections relate to changes in functional interactions (Song et al., 2015; Peng et al., 2023). Moreover, longitudinal studies involve repeated assessments over time to track changes in both structural and functional aspects of the brain, providing valuable insights into the recovery processes, neural reorganization, and the evolving impact of stroke over the course of rehabilitation (Desowska and Turner, 2019). Quantitative mapping of whole-brain connectivity at different scales illuminates the relationship between brain structure, function emergence, and the propagation of cellular damage causing functional deficits. Applying graph theory in network analysis categorizes connectivity changes, offers an insight into alterations associated with diverse neurological disorders (van den Heuvel and Sporns, 2019).

Pallast et al. (2020) applied diffusion network analysis in a mouse stroke model, providing the first experimental longitudinal mapping of cortical, subcortical and fiber tract changes. Using region-based diffusion tractography and graph analysis, the authors revealed a spontaneous functional improvement in mice after stroke (Pallast et al., 2020). Their findings did not reveal any reduced connectivity in homologous contralesional regions. However, decreased connectivity was confirmed between the ipsilesional motor cortex and the contralesional striatum, extending to the amygdala. Previous rat model research on photothrombosis indicated astroglial responses in the striatum up to 4 weeks post-stroke and transiently in the ipsilesional amygdala (Nowicka et al., 2008). Interestingly, Pallast et al. (2020) observed increased interhemispheric connectivity between homotopic motor areas, consistent with prior human and animal studies (Rehme and Grefkes, 2013; Pallast et al., 2020).

Other recent approaches for visualization of the tissue alterations utilize methods such as 3D histology (clearing techniques), serial 2D histology (connectivity atlases), and noninvasive *in vivo* imaging (dMRI) in rodents, primates, and humans (Van Essen et al., 2013; Oh et al., 2014; Calabrese et al., 2015). Clearing methods like CLARITY (Tomer et al., 2014; Epp et al., 2015) and 3DISCO/iDISCO (Pan et al., 2016) enable mapping in intact tissue. Alternatively, high-throughput tracing experiments and dMRI reveal connectivity and macroscopic changes associated with disease (Basser et al., 2000). These techniques generate large datasets requiring analytical tools for extracting neural network information. In a murine stroke model, Goubran et al. (2019) employed a pipeline, finding a strong link between MRI abnormalities and CLARITY-tissue staining at the microscopic level (Goubran et al., 2019). Bice et al. (2022) employed optogenetic techniques and optical imaging to investigate the effects of chronic optogenetic excitation in the contralesional hemisphere in a mice stroke photothrombotic model. The results revealed that contralesional excitation suppressed local cortical remapping and prevented the restoration of resting-state functional connectivity within the affected sensory-motor network. Importantly, the mice exposed to this excitation exhibited persistent limb-use asymmetry, accompanied by transcriptional changes in genes relevant to recovery. These findings emphasize the intricate connection between neural activity, circuit reconnection, and gene expression, underscoring the importance of targeted therapeutic interventions in stroke recovery (Bice et al., 2022).

### 8.3 Sensible selection of laboratory animals

Preclinical studies are not always easily translatable into clinical settings (Dhir et al., 2020) but the characteristics of the animals should be as close to the target population as possible. Therefore, the use of old animals would be ideal, yet this is not always the first choice in many studies (McCutcheon and Marinelli, 2009). Although most scientists claim to use adult mice, unfortunately, some studies were conducted on 6 – 10-week-old mice (Bacigaluppi et al., 2009; Gaire et al., 2019), when the brain is not fully developed, therefore the obtained results might be misleading. Moreover, a vast majority of animal experiments are carried out on males in order to avoid the influence of the estrous cycle (Braeuninger and Kleinschnitz, 2009). However, the impact of sex hormones on the experiments should not be overlooked, as estrogens were proven to exert protective effects in ischemia (see section “Estrogen administration”) (Braeuninger and Kleinschnitz, 2009).

### 8.4 Use of a clinically relevant ischemia model

The choice of the animal model influences the outcome a great deal. For example, Li et al. (2011) induced ischemia by two distinct methods, MCAO and cortical aspiration lesion (Li et al., 2011). The later model caused a significant decrease in the levels of zonula occludens-1 protein, the marker of tight junction integrity, in the ipsilateral thalamus, whereas MCAO left tight junctions intact. MCAO is a widely used method for the creation of focal ischemia. The distal version relies solely on the occlusion of the MCA, whereas proximal MCAO involves a transient or permanent blockade of carotid arteries. When performing the Koizumi method of proximal MCAO, the ipsilateral common carotid artery (CCA) is permanently ligated whereas in the case of the Longa method, the distal part of the external carotid artery (ECA) is dissected and the CCA is closed with a vessel clip during the time of the MCAO (Shahjouei et al., 2016). Since the brain depends solely on the blood supply brought by the two CCAs and the proximal MCAO involves the closure of one of them, it may be speculated as to whether this does not decrease the oxygenation in regions remote from the core and thus create false results. For instance, it was discovered that both Koizumi and Longa sham surgeries caused a significant increase in blood corticosterone 3 days after the intervention, but no changes in the remote hippocampus. They also found that the Koizumi method tends to activate the hypothalamic–pituitary–adrenal axis to a higher degree than that of Longa (Onufriev et al., 2021). Moreover, the coagulation of the ECA in the Longa method leads to necrosis of the head and neck muscles, which has a negative impact on the neurological functions of tested animals (Komatsu et al., 2021).

### 8.5 Accurate specification of the ischemic versus non-ischemic brain areas

As already stated above, the common locations of the ischemic core are known for each of the focal ischemia models. Nevertheless, every laboratory and even researcher performs the surgery slightly differently. For instance, the thickness of the filament used for MCAO impacts the lesion volume (Morris et al., 2016). Due to these small adaptations of the methods, it is necessary to describe the exact area of the primary injury, especially when we define the remote regions. Furthermore, when describing the histologic evaluation of a certain phenomenon (e.g., reduced expression of a certain protein), it is necessary to provide information about the location and type of cells that expressed it. Describing the results in general for the whole ipsilateral hemisphere may not be accurate, since each region of the hemisphere can show a different post-ischemic response.

### 8.6 Uniform terminology

When evaluating studies that reported the effect of focal ischemia on the brain tissue, one may encounter the use of varying terminology. The penumbra is by some researchers referred to as “peri-lesional area” (Witte et al., 2000; Jung et al., 2007; Sugimoto et al., 2014) or “peri-ischemic region” (Annunziato et al., 2013; Zhou et al., 2019; Formisano et al., 2020). On the other hand, “peri-ischemic tissue” may also characterize all the areas beyond the penumbra (Oermann et al., 2004; Gaire, 2022). The penumbra can be also called the “peri-infarct rim” and its border on the side closer to the ischemic core described as a demarcation zone (Hobohm et al., 2005). After the development of the post-ischemic tissue progressed and the glial scar was established, one publication also used the term “scar boundary zone” (Li et al., 2005). In one study, the penumbra was divided into the adjacent zone with a significantly reduced blood flow and the distant zone, in which perfusion was intact; the BBB permeability increased and cellular damage was present in both parts (Schoknecht et al., 2014). Moreover, sometimes the terms “core” and “penumbra” do not correspond to the actual places of the ischemic lesion and the thin apoptotic band around, respectively (Bona et al., 2019), which can then compromise the accuracy of the results. We can see that the use of terms is rather inconsistent, therefore, an agreement should be reached in order to prevent the misinterpretation of study outcomes.

### 8.7 The need for longitudinal studies

Post-ischemic reactions in remote regions are usually delayed and persist for a long time following the stroke. For this reason, studies about remote areas should cover the post-ischemic development for several weeks and assess the tissue both in subacute and chronic phases. As the changes in the remote areas may not be as pronounced as in the core or penumbra, the use of more sensitive markers is advisable [for example, Fluoro-Jade staining instead of NeuN antibody (Hosp et al., 2020)].

When the results from the ipsilateral hemisphere need to be compared to baseline values, it is a widely used practice to utilize the values acquired from the homotopic regions of the contralateral hemisphere. However, it is impossible to rely on the intactness of the contralateral hemisphere, as it is sometimes affected as well. In some cases, researchers consider the contralateral hemisphere a control, even though they detected significant alterations there. Thus, the correct way is to compare the results of the treated animals to those which underwent sham surgery.

## 9 Conclusion

Researchers in the field of stroke investigation are striving to uncover strategies that can prevent episodes of focal ischemia, enhance the quality of life for survivors, and mitigate premature mortality. To succeed in this effort, it is imperative to broaden our perspective beyond the confines of primary injury sites and adopt a holistic approach, viewing the brain as an interconnected system where all components influence each other. This involves scrutinizing the regions where damage may manifest subtly and over time.

Existing research underscores that ischemia exerts diverse effects beyond the penumbra, encompassing neurodegeneration, neuronal loss, disrupted synapses, hypertrophy of astrocytes and microglia, inflammatory responses, white matter degradation, extracellular matrix alterations, edema, amyloid plaques, and blood-brain barrier dysfunction. Notably, glial activation seems to be intricately linked to many of these alterations, as suggested by studies focused on the penumbral regions. However, only with the involvement of exo-focal structures we can have a comprehensive understanding of the ischemic mechanisms that is necessary for the development of innovative therapeutic approaches.

## Author contributions

LK: Conceptualization, Funding acquisition, Writing—original draft. MC: Validation, Writing—review and editing. ZA: Writing—review and editing. LV: Conceptualization, Resources, Supervision, Validation, Writing—review and editing.

## Figures and Tables

**Table:** 

Distal MCAO (the craniotomy model)	The distal MCAO model relies solely on the occlusion of the MCA. The MCA is exposed, and blood flow of the MCA is temporarily interrupted, for example by compression (tMCAO). Another possibility is to permanently close the blood flow in part of the MCA, by using bipolar cauterizing forceps (pMCAO).
Permanent MCAO (pMCAO)	A stroke model where obstruction blocking the MCA is not removed/blood flow is not restored. This model imitates patients who cannot have the clot removed (approximately 70 % of patients with large vessel stroke).
Photothrombosis	A method using intravascular photo-oxidation. An injected light-sensitive dye is irradiated with light through the exposed skull. This generates oxygen radicals that evoke platelet aggregation and thrombosis. Photothrombosis can produce ischemic injury in specific brain regions. This model can be permanent or transient (thrombus dissolves spontaneously), by modifying the conditions, such as light intensity.
Proximal MCAO (intraluminal suture MCAO model)	The proximal MCAO model is performed by inserting a suture into the internal carotid artery and advancing it until it blocks the origin of the MCA. There are 2 basic methods: the *Koizumi method* - the ipsilateral common carotid artery (CCA) is obstructed; and the *Longa method* - the distal part of the external carotid artery (ECA) is blocked. This method can be either transient (suture is removed) or permanent.
Transient MCAO (tMCAO)	In this type of stroke model, the obstruction is removed after a specific time, allowing a reperfusion. It simulates the situation in patients in which the clot was removed mechanically (e.g., endovascular thrombectomy) or by using drug administration.
