# Supplementary material for: Out of the core: the impact of focal ischemia in regions beyond the penumbra
Source: Front Cell Neurosci. 2024 Mar 5;18:1336886. doi: 10.3389/fncel.2024.1336886 (PMC10948541; doi:10.3389/fncel.2024.1336886)
Supplement: Supplementary file 1 [file Table_1.pdf]

ADCw	Apparent diffusion coefficient of water
APP	Amyloid precursor protein
ATP	Adenosine-triphosphate
BBB	Blood-brain barrier
BDNF	Brain-derived neurotrophic factor
BFGF	Basic fibroblast growth factor
BrdU	Bromodeoxyuridine
C3d	Complement component 3d
CA	Cornu ammonis
CC	Corpus callosum
CCA	Common carotid artery
CD68	Cluster of differentiation 68
CNS	Central nervous system
CSPGs	Chondroitin sulphate proteoglycans
CT	Computing tomography
CX43	Connexin 43
DFX	Deferoxamine
DTI	Diffusion tensor imaging
ECA	External carotid artery
ECM	Extracellular matrix
ECS	Extracellular space
E/T ratio	Estradiol/testosterone ratio
FGL	FG loop
GAP-43	Growth associated protein-43
GFAP	Glial fibrillary acidic protein
HA	Hyaluronan
HAS-2	Hyaluronan synthase-2
HIF-1	Hypoxia-inducible factor 1
HO-1	Heme oxygenase-1
Hyal	Hyaluronidase
Iba-1	Ionized calcium binding adaptor molecule 1
ICAM-1	Intercellular adhesion molecule 1
IL	Interleukin
INF	Interferon
LPA1	Lysophosphatidic acid receptor 1
MAG	Myelin-associated glycoprotein
MAP1LC3	Microtubule-associated protein 1A/1B-light chain 3
MAP2	Microtubule-associated protein 2
MBP	Myelin basic protein
MCA	Middle cerebral artery
MCAO	Middle cerebral artery occlussion
MCP-1	Monocyte chemoattractant protein-1
MMPs	Matrix metaloproteinases
MRI	Magnetic resonance imaging
NSCs	Neural stem cells
NEP1-40	Inhibitor against Nogo-A
NeuN	Neuronal nuclear protein
NF200	Neurofilament 200
NO	Nitric oxide
NOGOA	Neurite outgrowth inhibitor-A
NOS	Nitric oxide synthase

Nrf-2	Nuclear factor erythroid 2-related factor 2
P2X7	Purinergic receptor P2X7
PET	Positron emission tomography
pMCAo	Permanent Middle cerebral artery occlussion
PNNs	Perineuronal nets
PPAR- γ	Peroxisome proliferator-activated receptor γ
PUFAs	Polyunsaturated fatty acids
Ras	Rat sarcoma virus
Rhamm	Hyaluronan receptor
RhoA/ROCK	A/Rho-associated protein kinase
ROS	Reactive oxygen species
S100A10	S100 calcium-binding protein A10
SGZ	Subgranular zone
SN	Substantia nigra
SVZ	Subventricular zone
TGF-β	Transforming growth factor-β
tMCAO	Transient Middle cerebral artery occlusion
TNF-α	Tumor necrosis factor-α
VEGF	Vascular endothelial growth factor
VPN	Ventroposterior thalamic nucleus
WFA	Wisteria floribunda
WM	White matter
