# Supplementary material for: Out of the core: the impact of focal ischemia in regions beyond the penumbra
Source: Front Cell Neurosci. 2024 Mar 5;18:1336886. doi: 10.3389/fncel.2024.1336886 (PMC10948541; doi:10.3389/fncel.2024.1336886)
Supplement: Supplementary file 2 [file Table_2.PDF]

Supplementary Tab. 2 Overview of reactions in damaged tissue following focal ischemia in respective brain structures with focus on regional glial and neural response.

	Astrocytes	Microglia	Oligodendrocytes	NG2-glia	Neurons
**Cortex**	No changes at 24 h [1]<br>↑ GFAP immunostaining D4 & D7 [2]<br>↓ Cx43 on 1D & 3D, ↑ on D7 [3]	↑ number at D1 & D3 [4]<br>↑reactivity [1]	N/A	No changes at D1, D7 and D14 [5]	Degeneration [6, 7]<br>Intact neurons [1, 8]
**Hippocampus**	Astrogliosis from D1 [2, 3, 9-11]<br>Gradual ↑ in CX43⁺ cells [3]	↑ Iba-1 levels [10]<br>↑ cell density [11]<br>No activation [12]	↓ numbers in the W1 [10]	N/A	Degeneration after 12 h [13]<br>↓ numbers D3 – M12 [9, 10]<br>↓ numbers of cholinergic, NO^+ and NOS^+ neurons [14]<br>Constant numbers [11, 15]<br>↑ density of NSC in DG at D7 [16]
**Thalamus**	No changes at D1 [17]<br>Astrogliosis from D3 [17-21]<br>Astrocytic scar [22, 23]<br>No change in GFAP immunostaining for M2 [2]	Activation within D7 [12, 16, 18-22, 24-27]<br>↑ expression of microglial genes [21]<br>Concentration of iron in the cells [22]	N/A	↑ in number of $NG2^+$ and Iba-1^+ cells [24]	Damaged GABAergic neurons at D1 [28]<br>No changes on D1 [17]<br>↓ numbers of neurons [7, 19, 22, 24-27]<br>↑ in numbers of $nestin^+$ cells at W2 [25]
**Substantia nigra**	↑ size of the cell at D7 [17, 29]<br>↑ proliferation [19]<br>↑ intensity of GFAP immunostaining [30]<br>Normal-looking intracellular content [29]	↑ cell numbers and activation for M2[31, 32]<br>Swelling [30, 31]	No changes at W1 [29]	N/A	Aberrant intracellular content [29]<br>Healthy-looking neurons in W1 [17, 30]<br>↓ numbers of cells from D4 [29, 30]<br>↓ of dopaminergic neurons numbers [31-33]<br>Neuronophagia at W2 [30]<br>Release of TNF-α and IL-6 [17, 30]
**White matter**	↑ amount of GFAP at D7 [34]<br>↑ levels of C3d and LCN2 [34]<br>↓ S100A10 [34]	Activated cells at W1 [12]<br>No ameboid cells [35]	Damaged cells in CC at W8 [36]	N/A	$Nestin^+$ cells at W1 [24]
**Cerebellum**	N/A	No activated microglia at D28 [37]	N/A	N/A	No changes [38]
**Pons & midbrain**	N/A	↑ cell activation in between W2 – W26 [39, 40]	N/A	N/A	↓ numbers of dopaminergic neurons at M4 [33]
**Amygdala**	↑ GFAP immunostaining at D4 [2]	N/A	N/A	N/A	N/A
**VP & OT**	No changes at 24 h [1]	N/A	N/A	N/A	No changes at 24 h [1]
**Striatum**	N/A	N/A	Damaged cells (↓ MBP immunostaining) at D56 [36]	N/A	N/A
**Contra SN**	N/A	N/A	N/A	N/A	No changes at D14 [30]
**Contra striatum**	Cell swelling at D7 & D30 [41, 42]<br>↑ GFAP immunopositivity [41, 42]<br>Detached end-feet [41, 42]	Cell activation [41, 42]<br>↓ cell density at D1, D3 & D7 [6]	N/A	N/A	↓ cell density [41, 42]<br>Pyknotic cells [41, 42]<br>Neurodegeneration in W1 [6]<br>↓ content of NO in neurons at D1 and D3 [6]
**Contra cortex**	Abnormal intracellular content D7 & D30 [41, 42]<br>↑ GFAP immunopositivity [41, 42]<br>No astrogliosis [2]	Cell activation [41, 42]<br>↓ cell density at D7 [6]<br>No microgliosis [2]	N/A	N/A	↓ cell density [41, 42]<br>Pyknotic cells [41, 42]<br>Degeneration in W1[6]<br>↓ content of NO in cells at D1 and D3 [6]
**Contra HPP**	No astrogliosis [2]	No microgliosis [2]	N/A	N/A	No damage [10]
**Contra amygdala**	No astrogliosis [2]	No microgliosis [2]	N/A	N/A	N/A

Abbreviations: VP & OT = ventral pallidum and olfactory tubercle; GFAP = glial fibrillary acidic protein; CX43 = connexin 43; Iba-1 = ionized calcium-binding adaptor molecule-1; NSC = neuronal stem cells; DG = dentate gyrus; SOD = superoxide dismutase; NG2 = neuron-glial antigen 2; TNF-α = tumor necrosis factor α; IL-6 = interleukin 6; CC = corpus callosum; MBP = myelin basic protein; C3d = complement component 3; LCN2 = lipocalin 2; S100A10 = S100 calcium-binding protein A10; NO = nitric oxide.

Supplementary Table 2 (continuation). Overview of reactions in damaged tissue following focal ischemia in respective brain structures with focus on cell turnover, white matter and ECM.

	Cell turnover	White matter fibers	ECM	Miscellaneous
**Cortex**	No apoptotic cells at M1 [8]	Degeneration of axons and synapses at D7 [43]<br>↑ levels of GAP-43 [7]	↓ density in PNNs within several hours [8]<br>↓ Brevican immunostaining at D1 [8]<br>No changes in aggrecan and neurocan staining [8, 44]	Presence of HSP27 [45]
**Hippocampus**	↑ apoptotic rate at W2 [46]<br>↑ BrdU+ cells in the DG in W1 [47]	N/A	↓ of WFA-stainable PNNs [28]<br>↓ of immunoreactivity for type IV collagen [46]<br>↑ immunopositivity of MMP9 [46]	↑ amount of nNOS and iNOS [10]<br>↑ levels of SOD [10]<br>Larger HPP at M12 [11]
**Thalamus**	↑ in proliferation [25]	Degeneration of corticothalamic tracts [35]<br>↓ amount of GAP-43 [26]<br>↓ levels of SYN [7]	↓ WFA, aggrecan, neurocan and CSPG staining at D1 [20, 28]	↑ vascular density and ↑ blood flow [48]<br>↑ capillary thickness, ↑ proliferation of ECs [25]<br>↑ levels of BECN1 and LC3 [27]<br>APP plaques with iron [22, 23, 26, 49]<br>↑ immunoreactivity of HO-1 [22]<br>Atrophy of thalamus [50]<br>Microglia accumulated around APP plaques [22]
**Substantia nigra**	N/A	N/A	N/A	Shrinkage of SN at W1 [30]<br>↑ density of capillaries [48]<br>↑ tissue perfusion [48]
**White matter**	N/A	Demyelination in CC [34, 36]<br>↓ in MBP, MAG and NF200 [34]<br>↓ thickness of myelin fibres and their sheaths [34]<br>density of myelin fibres in CC unaffected at W4 [15]<br>Unchanged density of fibres in CC [51]<br>No abnormalities in IC at W2 [18]		
**Cerebellum**	↑ number of apoptotic cells at 24 h [52]<br>No apoptotic cells at D1 [53]<br>↑ number of apoptotic cells in the contralateral [54]	N/A	N/A	↓ ADC in both cerebella [52, 53]<br>Unchanged expression of genes related to apoptosis, hypoxia and ROS [54]<br>↑ expression of HO-1 and Nrf2 [54]
**Pons & midbrain**	N/A	N/A	N/A	N/A
**Amygdala**	N/A	N/A	N/A	N/A
**VP & OT**	N/A	N/A	N/A	N/A
**Striatum**	N/A	Demyelination (↑ SMI-32) [36]	N/A	↑ number of ECs, ↑ total length of blood vessels, ↑ number of blood vessels [36]
**Contra SN**	N/A	N/A	N/A	N/A
**Contra striatum**	N/A	↓ number of myelin sheaths [41, 42]	N/A	Damaged BBB [41, 42]
**Contra cortex**	N/A	↓ number of myelin sheaths [41, 42]	↓ density of PNNs at M1 [8]<br>↑ levels of neurocan at D2 & D4 [44]<br>↑ CSPG expression D3 – D14 [55]	Damaged BBB [41, 42]
**Contra HPP**	↑ number of BrdU+ cells in DG at W1 [47]	N/A	N/A	N/A

**Contra amygdala**	N/A	N/A	N/A	N/A

Abbreviations: VP & OT = ventral pallidum and olfactory tubercle; GAP-43 = growth-associated protein 43; PNNs = perineuronal nets; HSP27 = heat shock protein 27; DG = dentate gyrus; BrdU = bromodeoxyuridine; WFA = *Wisteria floribunda* agglutinin; MMP9 = metalloproteinase 9; nNOS = neuronal nitric oxide synthase; iNOS = inducible nitric oxide synthase; APP = amyloid precursor protein; SYN = synapsin; CSPG = chondroitin sulphate proteoglycan; ECs = endothelial cells; BECN1 = beclin 1; LC3 = microtubule-associated protein 1A/1B-light chain 3; HO-1 = heme oxidase 1; CC = corpus callosum; MBP = myelin basic protein; MAG = myelin-associated glycoprotein; NF200 = neurofilament 200; IC = internal capsule; ADC = apparent diffusion coefficient; ROS = reactive oxygen species; Nrf2 = nuclear factor erythroid 2–related factor 2; SMI-32 = Sternberger monoclonal incorporated antibody 32; BBB = blood brain barrier.

1. Melani, A., et al., *P2X7 receptor modulation on microglial cells and reduction of brain infarct caused by middle cerebral artery occlusion in rat.* J Cereb Blood Flow Metab, 2006. **26**(7): p. 974-82.
2. Nowicka, D., et al., *Spatiotemporal dynamics of astroglial and microglial responses after photothrombotic stroke in the rat brain.* Acta Neurobiol Exp (Wars), 2008. **68**(2): p. 155-68.
3. Haupt, C., O.W. Witte, and C. Frahm, *Temporal profile of connexin 43 expression after photothrombotic lesion in rat brain.* Neuroscience, 2007. **144**(2): p. 562-70.
4. Gaire, B.P., et al., *Lysophosphatidic acid receptor 1 (LPA(1)) plays critical roles in microglial activation and brain damage after transient focal cerebral ischemia.* J Neuroinflammation, 2019. **16**(1): p. 170.
5. Tanaka, K., et al., *Activation of NG2-positive oligodendrocyte progenitor cells during post-ischemic reperfusion in the rat brain.* Neuroreport, 2001. **12**(10): p. 2169-74.
6. Bona, M., et al., *Response of distant regions affected by diaschisis commissuralis in one of the most common models of transient focal ischemia in rats.* J Chem Neuroanat, 2019. **101**: p. 101666.
7. Chen, X.R., et al., *Neuroprotective effect of chondroitinase ABC on primary and secondary brain injury after stroke in hypertensive rats.* Brain Res, 2014. **1543**: p. 324-33.
8. Karetko-Sysa, M., J. Skangiel-Kramska, and D. Nowicka, *Disturbance of perineuronal nets in the perilesional area after photothrombosis is not associated with neuronal death.* Exp Neurol, 2011. **231**(1): p. 113-26.
9. Ouyang, F., et al., *Neuronal loss without amyloid-beta deposits in the thalamus and hippocampus in the late period after middle cerebral artery occlusion in cynomolgus monkeys.* Brain Pathol, 2020. **30**(1): p. 165-178.
10. Uchida, H., et al., *Damage to neurons and oligodendrocytes in the hippocampal CA1 sector after transient focal ischemia in rats.* Cell Mol Neurobiol, 2010. **30**(7): p. 1125-34.
11. Brait, V.H., et al., *Longitudinal hippocampal volumetric changes in mice following brain infarction.* Sci Rep, 2021. **11**(1): p. 10269.
12. Schroeter, M., S. Jander, and G. Stoll, *Non-invasive induction of focal cerebral ischemia in mice by photothrombosis of cortical microvessels: characterization of inflammatory responses.* J Neurosci Methods, 2002. **117**(1): p. 43-9.
13. Butler, T.L., et al., *Neurodegeneration in the rat hippocampus and striatum after middle cerebral artery occlusion.* Brain Res, 2002. **929**(2): p. 252-60.
14. Park, H.J., et al., *The Protective Effect of Black Ginseng Against Transient Focal Ischemia-induced Neuronal Damage in Rats.* Korean J Physiol Pharmacol, 2011. **15**(6): p. 333-8.
15. Zhou, J., et al., *Long-term post-stroke changes include myelin loss, specific deficits in sensory and motor behaviors and complex cognitive impairment detected using active place avoidance.* PLoS One, 2013. **8**(3): p. e57503.
16. Klein, R., et al., *The Neural Cell Adhesion Molecule-Derived (NCAM)-Peptide FG Loop (FGL) Mobilizes Endogenous Neural Stem Cells and Promotes Endogenous Regenerative Capacity after Stroke.* J Neuroimmune Pharmacol, 2016. **11**(4): p. 708-720.

17. Loos, M., M. Dihne, and F. Block, *Tumor necrosis factor-alpha expression in areas of remote degeneration following middle cerebral artery occlusion of the rat.* Neuroscience, 2003. **122**(2): p. 373-80.
18. Hirouchi, Y., et al., *Neuroimaging and histopathological evaluation of delayed neurological damage produced by artificial occlusion of the middle cerebral artery in Cynomolgus monkeys: establishment of a monkey model for delayed cerebral ischemia.* Exp Toxicol Pathol, 2007. **59**(1): p. 9-16.
19. Hobohm, C., et al., *Decomposition and long-lasting downregulation of extracellular matrix in perineuronal nets induced by focal cerebral ischemia in rats.* J Neurosci Res, 2005. **80**(4): p. 539-48.
20. Ladwig, A., et al., *Osteopontin Attenuates Secondary Neurodegeneration in the Thalamus after Experimental Stroke.* J Neuroimmune Pharmacol, 2019. **14**(2): p. 295-311.
21. Justicia, C., P. Ramos-Cabrer, and M. Hoehn, *MRI detection of secondary damage after stroke: chronic iron accumulation in the thalamus of the rat brain.* Stroke, 2008. **39**(5): p. 1541-7.
22. Dihne, M., et al., *Different mechanisms of secondary neuronal damage in thalamic nuclei after focal cerebral ischemia in rats.* Stroke, 2002. **33**(12): p. 3006-11.
23. van Groen, T., et al., *Transformation of diffuse beta-amyloid precursor protein and beta-amyloid deposits to plaques in the thalamus after transient occlusion of the middle cerebral artery in rats.* Stroke, 2005. **36**(7): p. 1551-6.
24. Ling, L., et al., *Neurogenesis and angiogenesis within the ipsilateral thalamus with secondary damage after focal cortical infarction in hypertensive rats.* J Cereb Blood Flow Metab, 2009. **29**(9): p. 1538-46.
25. Wang, F., et al., *Nogo-A is involved in secondary axonal degeneration of thalamus in hypertensive rats with focal cortical infarction.* Neurosci Lett, 2007. **417**(3): p. 255-60.
26. Xu, W., et al., *Blockade of Nogo-A/Nogo-66 receptor 1 (NgR1) Inhibits Autophagic Activation and Prevents Secondary Neuronal Damage in the Thalamus after Focal Cerebral Infarction in Hypertensive Rats.* Neuroscience, 2020. **431**: p. 103-114.
27. Cao, Z., et al., *Unique Subtype of Microglia in Degenerative Thalamus After Cortical Stroke.* Stroke, 2021. **52**(2): p. 687-698.
28. Hartig, W., et al., *Abolished perineuronal nets and altered parvalbumin-immunoreactivity in the nucleus reticularis thalami of wildtype and 3xTg mice after experimental stroke.* Neuroscience, 2016. **337**: p. 66-87.
29. Zhao, F., et al., *Ultrastructural and MRI study of the substantia nigra evolving exofocal post-ischemic neuronal death in the rat.* Neuropathology, 2002. **22**(3): p. 91-105.
30. Dihne, M. and F. Block, *Focal ischemia induces transient expression of IL-6 in the substantia nigra pars reticulata.* Brain Res, 2001. **889**(1-2): p. 165-73.
31. Huh, Y., et al., *Microglial activation and tyrosine hydroxylase immunoreactivity in the substantia nigral region following transient focal ischemia in rats.* Neurosci Lett, 2003. **349**(1): p. 63-7.
32. Prinz, V., et al., *MRI heralds secondary nigral lesion after brain ischemia in mice: a secondary time window for neuroprotection.* J Cereb Blood Flow Metab, 2015. **35**(12): p. 1903-9.
33. Kronenberg, G., et al., *Exofocal dopaminergic degeneration as antidepressant target in mouse model of poststroke depression.* Biol Psychiatry, 2012. **72**(4): p. 273-81.
34. Wan, T., et al., *Astrocytic phagocytosis contributes to demyelination after focal cortical ischemia in mice.* Nat Commun, 2022. **13**(1): p. 1134.
35. Reichmann, G., et al., *Dendritic cells and dendritic-like microglia in focal cortical ischemia of the mouse brain.* J Neuroimmunol, 2002. **129**(1-2): p. 125-32.
36. Cai, M., et al., *Promoting Neurovascular Recovery in Aged Mice after Ischemic Stroke - Prophylactic Effect of Omega-3 Polyunsaturated Fatty Acids.* Aging Dis, 2017. **8**(5): p. 531-545.
37. Gerhard, A., et al., *Evolution of microglial activation in patients after ischemic stroke: a [11C](R)-PK11195 PET study.* Neuroimage, 2005. **24**(2): p. 591-5.
38. Yang, G., B.B. Rothrauff, and R.S. Tuan, *Tendon and ligament regeneration and repair: clinical relevance and developmental paradigm.* Birth Defects Res C Embryo Today, 2013. **99**(3): p. 203-222.
39. Thiel, A., et al., *The temporal dynamics of poststroke neuroinflammation: a longitudinal diffusion tensor imaging-guided PET study with 11C-PK11195 in acute subcortical stroke.* J Nucl Med, 2010. **51**(9): p. 1404-12.

40. Radlinska, B.A., et al., *Multimodal microglia imaging of fiber tracts in acute subcortical stroke.* Ann Neurol, 2009. **66**(6): p. 825-32.
41. Garbuzova-Davis, S., et al., *Compromised blood-brain barrier competence in remote brain areas in ischemic stroke rats at the chronic stage.* J Comp Neurol, 2014. **522**(13): p. 3120-37.
42. Garbuzova-Davis, S., et al., *Blood-brain barrier alterations provide evidence of subacute diaschisis in an ischemic stroke rat model.* PLoS One, 2013. **8**(5): p. e63553.
43. Lee, M.C., et al., *Ultrastructural Dendritic Changes Underlying Diaschisis After Capsular Infarct.* J Neuropathol Exp Neurol, 2020. **79**(5): p. 508-517.
44. Deguchi, K., et al., *Expression of neurocan after transient middle cerebral artery occlusion in adult rat brain.* Brain Res, 2005. **1037**(1-2): p. 194-9.
45. Popp, A., et al., *Identification of ischemic regions in a rat model of stroke.* PLoS One, 2009. **4**(3): p. e4764.
46. Yang, P., et al., *Sijunzi decoction may decrease apoptosis via stabilization of the extracellular matrix following cerebral ischaemia-reperfusion in rats.* Exp Ther Med, 2019. **18**(4): p. 2805-2812.
47. Takasawa, K., et al., *Increased proliferation of neural progenitor cells but reduced survival of newborn cells in the contralateral hippocampus after focal cerebral ischemia in rats.* J Cereb Blood Flow Metab, 2002. **22**(3): p. 299-307.
48. Yanev, P., et al., *Magnetic resonance imaging of local and remote vascular remodelling after experimental stroke.* J Cereb Blood Flow Metab, 2017. **37**(8): p. 2768-2779.
49. Lipsanen, A., M. Hiltunen, and J. Jolkkonen, *Chronic ibuprofen treatment does not affect the secondary pathology in the thalamus or improve behavioral outcome in middle cerebral artery occlusion rats.* Pharmacol Biochem Behav, 2011. **99**(3): p. 468-74.
50. Arlicot, N., et al., *Detection and quantification of remote microglial activation in rodent models of focal ischaemia using the TSPO radioligand CLINDE.* Eur J Nucl Med Mol Imaging, 2010. **37**(12): p. 2371-80.
51. Reitmeir, R., et al., *Post-acute delivery of erythropoietin induces stroke recovery by promoting perilesional tissue remodelling and contralesional pyramidal tract plasticity.* Brain, 2011. **134**(Pt 1): p. 84-99.
52. Ma, Z., et al., *Evaluation of crossed cerebellar diaschisis after cerebral infarction in MCAO rats based on DKI.* Eur J Clin Invest, 2022. **52**(4): p. e13716.
53. Yang, Y., et al., *Apparent diffusion coefficient evaluation for secondary changes in the cerebellum of rats after middle cerebral artery occlusion.* Neural Regen Res, 2013. **8**(31): p. 2942-50.
54. Kidani, N., et al., *Cerebellar Blood Flow and Gene Expression in Crossed Cerebellar Diaschisis after Transient Middle Cerebral Artery Occlusion in Rats.* Int J Mol Sci, 2020. **21**(11).
55. Liu, Z., et al., *Beneficial effects of gfap/vimentin reactive astrocytes for axonal remodeling and motor behavioral recovery in mice after stroke.* Glia, 2014. **62**(12): p. 2022-33.
